# Estimating the extent of horizontal gene transfer in metagenomic sequences

**DOI:** 10.1186/1471-2164-9-136

**Published:** 2008-03-24

**Authors:** Javier Tamames, Andrés Moya

**Affiliations:** 1 Instituto Cavanilles de Biodiversidad y Biología Evolutiva. Universidad de Valencia. Polígono La Coma s/n, 46980 Paterna (Valencia), Spain; 2 CIBER en Epidemiología y Salud Pública (CIBER-ESP), Spain

## Abstract

**Background:**

Although the extent of horizontal gene transfer (HGT) in complete genomes has been widely studied, its influence in the evolution of natural communities of prokaryotes remains unknown. The availability of metagenomic sequences allows us to address the study of global patterns of prokaryotic evolution in samples from natural communities. However, the methods that have been commonly used for the study of HGT are not suitable for metagenomic samples. Therefore it is important to develop new methods or to adapt existing ones to be used with metagenomic sequences.

**Results:**

We have created two different methods that are suitable for the study of HGT in metagenomic samples. The methods are based on phylogenetic and DNA compositional approaches, and have allowed us to assess the extent of possible HGT events in metagenomes for the first time. The methods are shown to be compatible and quite precise, although they probably underestimate the number of possible events. Our results show that the phylogenetic method detects HGT in between 0.8% and 1.5% of the sequences, while DNA compositional methods identify putative HGT in between 2% and 8% of the sequences. These ranges are very similar to these found in complete genomes by related approaches. Both methods act with a different sensitivity since they probably target HGT events of different ages: the compositional method mostly identifies recent transfers, while the phylogenetic is more suitable for the detections of older events. Nevertheless, the study of the number of HGT events in metagenomic sequences from different communities shows a consistent trend for both methods: the lower amount is found for the sequences of the Sargasso Sea metagenome, while the higher quantity is found in the whale fall metagenome from the bottom of the ocean. The significance of these observations is discussed.

**Conclusion:**

The computational approaches that are used to find possible HGT events in complete genomes can be adapted to work with metagenomic samples, where a level of high performance is shown in different metagenomic samples. The percentage of possible HGT events that were observed is close to that found for complete genomes, and different microbiomes show diverse ratios of putative HGT events. This is probably related with both environmental factors and the composition in the species of each particular community.

## Background

Horizontal gene transfer (HGT) is believed to be a very important phenomenon in prokaryotic evolution, as it enables the acquisition of new genes or sets of genes that can accelerate evolution and adaptation to new environments or changing conditions.

In recent years, the availability of complete genome sequences from a wide range of prokaryotic species has made it possible to find many instances of potentially transferred genes in different genomes [1]. Nevertheless, the actual extent and importance of these events, and therefore the relevance of HGT in the evolution of prokaryotes, is still a matter of debate [1-6].

HGT has been extensively studied for sequenced genomes, but its real extent in natural populations in different environments remains unknown. Many of these communities are interesting from the ecological, biotechnological and/or clinical points of view, and therefore it is very important to understand how their members interact, evolve and innovate. HGT can be a very relevant factor since it may influence the dynamics of a community, and a recent report emphasises its important contribution to niche specialisation and adaptation [7]. However, the real impact of HGT in natural populations is unknown, and to date we do not know whether these events preferentially influence communities in particular environments. The advent of metagenomic sequencing [8,9] provides enough environmental DNA sequences to address the study of global patterns of prokaryotic evolution in natural communities, including HGT.

Two types of methods have been used to detect HGT events in genomic sequences: Phylogenetic methods, based on the examination of the phylogenies of individual genes or proteins; and compositional methods, based on the analysis of DNA composition, which is assumed to contain some evolutionary information in the form of species-specific signatures.

In theory, phylogenetic analysis is a powerful tool used to recognize HGT events. On building the phylogenetic tree of a family of sequences from different species, putative HGT can be identified when the position of a specie in the tree does not match that of a reference phylogeny, which can be obtained in different ways: from universally conserved genes such as 16S rRNA or some tRNA synthetases, or by supertrees of conserved genes merged together [10]. Phylogenetic methods are therefore usable with individual genes, as long as a set of orthologues is known. In the practice, phylogenies are often noisy and/or inconclusive for several reasons (lack of homologues for specific genes, phylogenetic artefacts such as long-branch attraction, inclusion of paralogues, uneven mutation rates, etc). Therefore, the results of a phylogenetic analysis must be carefully examined before proposing HGT. This task is more difficult for metagenomic sequences because the origin of the sequence is almost always unknown (since the metagenome is a mixture of sequences from different species) and, consequently, the construction of a reference tree is not straightforward. Nevertheless, at least one attempt has been made to study environmental sequences by means of the comparison of individual phylogenies [11], which must be taken as a valuable proof of concept.

Several authors have used these phylogenetic approaches to detect and quantify HGT in genomes, orthologous groups of genes and protein families [12-16]. These methods estimate a rather low extent of HGT in all cases, usually below 2%. A possible explanation for this is that, since the barriers to HGT seem much easier to overcome when closely related species are involved [17], it is likely that most HGT events are produced between species belonging to the same taxon. These events are much more difficult to detect using phylogenetic analysis as the resolution of the trees decreases for such a small range. Consequently, it is likely that specific HGT events cannot be conclusively determined using phylogenetic approaches, leading to an underestimation in the frequency of HGT.

Compositional methods are based on the assumption that individual genomes have characteristic features regarding their DNA composition, which has been termed DNA or genomic signatures. Each genome has its own pattern of GC content and codon usage due to a combination of environmental and genetic factors [18-20]. Recently transferred regions can be detected since they possess a different DNA signature to that of the host genome which reflect their foreign origin [21-23]. Therefore, HGT events in complete genomes could be detected by recognising zones of different or unusual compositional patterns. In these cases, the genomic signature will be closer to those of the donor genomes, which in some cases can reveal the phylogenetic origin. As time passes, the foreign sequence is shaped by the biases in the replication and reparation machinery, which causes its signature to progressively resemble that of the host. This process is known as amelioration, and implies that the original sequence can no longer be recognised as that from a foreign origin on the basis of nucleotide composition alone [24]. Therefore, these methods perform better to detect recent HGT events, where genomic signatures differ significantly between the donor and recipient genomes.

Many compositional methods based on several approaches and algorithms exist [21-23,25-29]. The percentage of HGT estimated by compositional methods is significantly higher than by methods based on phylogenies. In addition, different methods have been shown to predict very different sets of genes as having been horizontally transferred [28,30-32]. This apparently discouraging result has been recently interpreted to mean that each method is tuned to detect gene transfers of different ages [32]. Normally, compositional methods have been used to analyse large genomic sequences since a significant amount of sequence is needed to extract the global features. This is a serious drawback for its use with metagenomic sequences.

Both the phylogenetic and compositional approaches have their advantages and drawbacks, even though the two are complementary since they use independent sets of information. They make predictions based on different features and can, therefore, be combined to provide a more comprehensive insight into the relevance of HGT processes.

Here, we propose a double approach to measure the incidence of HGT in short sequences of unknown origin as these are obtained by metagenomic sequencing of natural prokaryotic communities. Both a compositional and phylogenetic method were derived and tested in order to apply them to complex mixtures of sequences. The methods were then used to study the sequences of several available metagenomic projects.

## Methods

### Obtaining sequences

Several metagenomic projects have deposited DNA sequences of contigs in the GenBank database. The metagenomes analysed in this work are representative of different environments. Two of them are from marine locations: whale fall, the prokaryotic community living in whale carcasses at the bottom of the sea (28,151 contigs) [33], and the Sargasso Sea metagenome from surface sea water samples (811,372 contigs) [34], representing a planktonic community. Another is terrestrial, sampled from the soil of a farm (135,347 contigs) [33], and the last is a host-associated community, the human gut microbiome (10,488 contigs) [35]. The average length of the contigs is close to 1 Kb for all metagenomes (see Additional file 1).

Due to the intensive computational resources and the time needed to completely analyse the larger metagenomes (Farm soil and Sargasso Sea), we only worked with a representative fraction of these which consisted in 60,000 contigs randomly selected from these metagenomes.

### Generating random metagenomes

To benchmark the ability of the phylogenetic method to accurately assign taxa to the sequences, we created artificial metagenomes taking random sub-sequences from completely sequenced genomes. Sequences of 3000 bps were selected from random positions of the approximately 400 complete genomes available when the study was performed, while overlapping sequences were discarded. The complete taxonomy for the genomes was also retrieved to be used in the automatic evaluation of the results. Genomes and taxonomy were downloaded from NCBI [36]. The original species and taxa for the random metagenome referenced in the text show a very heterogeneous composition, and are seen in Additional file 2. The goodness of the assignments was estimated by means of the precision and recall measures (Precision = TP/TP+FP; Recall = TP/TP+FN, where TP: true positives; FP: false positives; FN: false negatives).

### Data set for rare taxa

To evaluate method performance when poorly studied taxa are present, a new data set was generated. We focused on taxa having at least one fully-sequenced genome (to extract sequences from it) and a low number of sequences in the GenBank non-redundant protein database [37] of March 2007 (to increase the likelihood of not finding close homologues). We selected three taxa at the 'class' taxonomic rank for the test: The bacterial Planctomycetacia (Bacteria/Planctomycetes/Planctomycetacia), with 31753 sequences in the GenBank database and one sequenced genome (*Rhodopirellula baltica*); also the bacterial Dehalococcoidetes (Bacteria/Chloroflexi/Dehalococcoidetes) with 9122 sequences and two closely related sequenced genomes (*Dehalococcoides ethanogenes *and *Dehalococcoides sp.*); and the archaeal Methanopyri (Archaea/Euryarcheota/Methanopyri), with 3847 sequences and one sequenced genome (*Methanopyrus kandleri*). A test data set was created, comprising 100 sequences of 3000 nucleotides long for each taxon, which were randomly extracted from the complete genomes belonging to these taxa.

### Analysing contigs

To find putative ORFs, we performed homology searches for each contig in the metagenome, by means of Blastx runs against the non-redundant GenBank protein database. Only prokaryotic hits with e-values below 1e-04 were considered. Identical hits were found when benchmarking with data sets derived from complete genomes since the corresponding proteins have already been deposited in GenBank. This artificially eases the assignment to produce an inaccurate measure of method performance. When benchmarking, we removed all hits from the same species as the original sequence (including strains) to match the situation in which the identical sequence is unknown.

Query and hit must align in at least 50% of the full length of both proteins. In this way, partial hits that could correspond to, for instance, single domains, are discarded. An exception is made when the hit is located on the extreme of the contig, and is truncated because of this. In this case, it is required to find one end of the protein. If the hit is interrupted by both ends of the contig, the alignment must span the full length of it. All hits not fulfilling these criteria are discarded.

After this step, the hits occurring in the same position and frame are considered homologues. We consider that two hits are in the same position if they overlap by at least 75%. Note that according to this definition, two hits that overlap completely but where one is much shorter than the other, will not be recognised as the same hit. The smaller hit is considered a fragment and is, therefore, dismissed. After grouping this step, the remaining hits or groups of homologue hits identify the putative ORFs in the contig, that are functionally characterised by means of Blast homology searches against the COG database [38]. A depiction of the procedure can be seen in Additional file 3.

## Detection of HGT

We have developed two different methods to detect possible HGT events, both of which aim to find transitions in the DNA sequences. The first is a phylogenetic method that relies on the taxonomic assignment of the ORFs found in the contig to identify the instances of ORFs assigned to disparate taxa (related approaches can be found in [39-41]). The second is a compositional method that scans DNA, and looks for zones of differential oligonucleotide usage. We now go on to describe both methods in detail.

### Phylogenetic assignment method

For the homologues found for a given putative ORF, a distance score is calculated as the inverse of the bit score of their alignment. The taxonomy for each homologue is obtained from the GenBank database and chopped to the desired taxonomic rank (in decreasing order: kingdom (first rank), phylum (second), class (third), order (forth) and family (fifth)). When more than one homologue is found for the same species, only the best hit is kept, thus effectively excluding paralogues. The ORF is assigned to a given taxon if the lowest distance scores are those that correspond to the homologues from it. Only two homologues from other taxa are allowed to have a lower score. If this is not the case, we often find that the ORF has many close homologues from a single taxon, in addition to several others from different taxa. This could be the consequence, for instance, of a high degree of transfers in that particular protein family. In such cases, the ORF is assigned to the taxon whose average distance score is at least 15% lower than that of any other. Examples of this procedure are depicted in Additional file 4.

In any case, an ORF requires at least three homologues with the taxon to be assigned to it. To avoid assignments when only distant homologues are found, a minimum identity is required between the ORF and the homologues (a default value is 30% of identity), and a minimum distance score is also needed. All homologues below these levels are not considered.

If the ORF could not be assigned by this procedure, a list of candidate taxa is kept for it, containing the average distance scores for all taxa for which at least one homologue has been found. This list will be subsequently used to improve the assignments.

**Figure 1:**
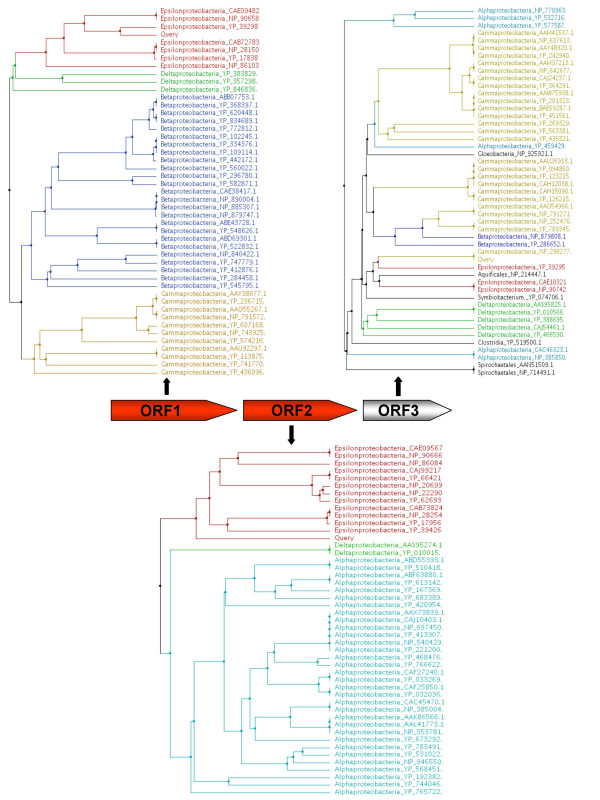
**Example of the assignment of a contig.** The phylogenetic tree including the homologues found for each of the putative ORFs is shown, and coloured according the taxa. According to the phylogenetic method, the first two ORFs could be assigned to the Epsilon-proteobacteria class, while the third remains unassigned. Since the majority of the ORFs in the contig belong to Epsilon-proteobacteria, this would be the proposed source for the contig itself.

Usually, between 10–40% of the ORFs of real metagenomes can be accurately assigned in this way. It is possible to take advantage of the fact that the contigs often contain more than one ORF to increase the number of assignments while maintaining a very high accuracy. For instance, let us suppose that a contig contains three ORFs, and we can clearly assign a common taxon for two of them. If the third ORF could not be assigned, we could assume that it belongs to the same taxon, provided it has close homologues from it (Figure 1). Therefore, we have introduced an additional stage that takes into account the assignments for the neighbouring ORFs. The unassigned ORF is scored in the following way: the first five taxa in the list of candidate taxa are scored with 1/*r*, where *r *is their rank in that list. The scores of the neighbouring ORFs are then added to this. If the neighbouring ORF has already been assigned to a given taxon, a score of one is added only to that taxon. An ORF is assigned to the taxon with the highest score, given that this is higher than 1.5. For instance, a frequent scenario is that of an unassigned ORF having both its closest homologue and a neighbouring ORF belonging to a given taxon. In this case, the final score for that taxon will be equal to two, and the ORF will be assigned to it. An ORF cannot be assigned to a taxon that it is not in its candidate list, which implies that the ORF must have at least one homolog belonging to the taxon.

This procedure increases the number of assignments in 10–12% for random metagenomes, and precision remains unaltered. For real metagenomes, the increase in the number of assignments depends greatly on the average number of ORFs in the contigs (see Additional file 1), which ranges between 3% for soil and 20% for Sargasso sea metagenomes.

**Figure 2:**
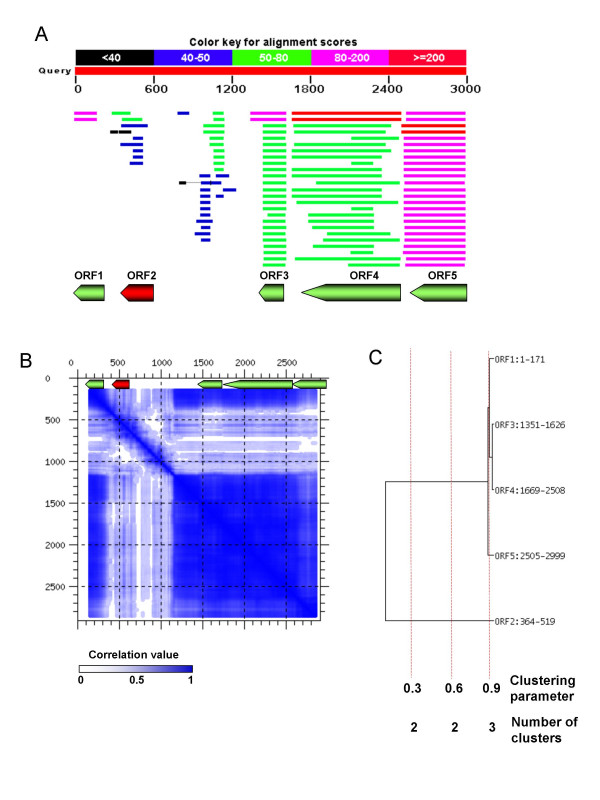
**A contig of *Fusobacterium nucleatum *shows a potential HGT event found by phylogenetic assignment and is supported by the compositional method.** A) Five ORFs have been clearly detected (a sixth ORF could exist, but it has not been considered since the homology search results in partial, short matches). Four of these ORFs (shown in green) have been assigned to *Fusobacterium *taxa (Fusobacteriaceae). The best hit for the second ORF (shown in red) is a short protein found in *Vibrio vulnificus *(Gamma-proteobacteria), and no hits with Fusobacteriales have been found in that zone. This can be indicative of a putative foreign origin of that ORF. B) The zone also shows atypical composition, which is very apparent and easily seen in the compositional diagram. C) Clustering of the distance matrix of compositional correlations. A clustering parameter indicates the point at which the groups of compositionally similar ORFs are extracted. The lower the value, the fewer the transitions found, since rather dissimilar ORFs are allowed to fall into the same cluster. In this example, the DNA composition of ORF 2 is so different that it will be recognised as being atypical at all values of the clustering parameter.

Once the ORFs have been assigned in this way, a possible HGT event will be predicted when a contig contains sequences from different taxa (Figure 2A). Unassigned ORFs are not considered.

In addition, a contig is assigned to a particular taxon if 75% of its ORFs, or more, belong to it, which can be used to study the taxonomic composition of metagenomes.

### Compositional method

Standard compositional methods for detecting HGT rely on detecting unusual characteristics in the compositional profile of the sub-sequences in comparison with a reference profile, normally that of the whole genome. The compositional profiles are usually expressed as a measure of the frequency of the constituent oligonucleotides. These standard methods cannot be readily used in this scenario since the sequences are too short to derive a reference profile. Therefore, we have followed a different approach, which works as follows: we count all oligonucleotides of size *n *for each putative ORF, using a sliding window of that size, which is moved through the sequence. The best results are achieved when *n *= 4 (tetranucleotides). The procedure creates a compositional vector for each ORF, containing the frequencies of all possible oligonucleotides. Complementary oligonucleotides are also included to eliminate any strand bias. The ORFs are then compared to each other by calculating Pearson's correlation coefficient between their compositional vectors. A low correlation coefficient indicates a dissimilar composition for these two ORFs. The correlation values between all ORFs in the contig are used to create a distance matrix, which is hierarchically clustered using the Cluster v2.11 program [42]. The resulting tree reflects the similarities between the DNA compositions of the ORFs in the contig (Figure 2C). The clusters of sequences are obtained by chopping the tree at a fixed correlation value.

When only one cluster is obtained, all ORFs in the contig are compositionally similar, and there is no indication of HGT. Otherwise, the contig shows compositional transitions that can be the result of HGT events. The correlation value at which clusters are extracted, which we will refer to as the "clustering parameter", is critical for the performance of the method. When it is set at low values, a high divergence in composition is needed to fall below the threshold. Therefore, we obtain few transitions, but these are very reliable in terms of compositional dissimilarity. On the other hand, using high values of the clustering parameter yields many possible transitions, but diminishes reliability (Additional file 2).

### Compositional diagrams

We have created a way to depict the DNA composition of a given sequence for visually inspecting the presence of compositional transitions. These compositional diagrams are generated in the following way: for a given DNA sequence, sub-sequences of a given length (by default, 300 base pairs) are extracted, starting in the first position of the sequence and advancing ten nucleotides each time. Therefore, each sub-sequence overlaps the previous one by 290 base-pairs. As before, we create a compositional vector for each sub-sequence which contains their oligonucleotide frequencies (tetranucleotides, by default). Then all pairs of vectors are compared by calculating their Pearson's correlation coefficient. The correlation values are transformed into colour intensity (higher correlations correspond to more intense colours), which are shown in a dot plot in the coordinates corresponding to the central position of both sub-sequences (Figure 2B and Additional file 5). In this way, a sharp transition in DNA composition is clearly visible as a zone of light colours (corresponding to low correlations) in the diagram.

### Benchmarking the compositional method on the *Escherichia coli *genome

To assess the accuracy of the compositional method in detecting transitions that could correspond to HGT events, we used the *E. coli *K-12 complete genome because many different methods have provided HGT predictions for it. We created artificial contigs, segments of the *E. coli *genome containing four consecutive ORFs. Contigs were obtained by sliding a window, ORF by ORF, through the complete genome. ORFs shorter than 250 base pairs were excluded. In this way, we obtained 3998 artificial contigs. For these, we counted the number of methods that predict HGT for each ORFs contained in the contig, according to the compilation of compositional HGT predictions by Dufraigne and colleagues [28] that gathers the results for six different methods. We considered that it contains a compositional transition (and a probable HGT boundary) if consecutive ORFs differ by two or more in the number of methods predicting it as HGT. For instance, if a ORF is not predicted as being transferred by any method, but the next ORF is predicted by two methods or more, the contig is considered to contain a compositional transition that can correspond to the boundary of an HGT event. The highest value of the differences in predictions for consecutive ORFs in the contig is called "difference in methods" (for instance, it is equal to two in the example above). In this way, 1448 contigs are predicted to contain a boundary of a putative HGT event (which would assume a minimum of 362 possible HGT events in the genome of *E. coli *since the same boundary will appear in four consecutive contigs).

## Results and Discussion

### Taxonomic assignment method

#### Benchmarking

To assess the accuracy of the results produced by the taxonomic assignment method, we created data sets composed of sequences taken randomly from complete genomes. In this way, the assignment is easy to check since the original taxon is known. Those cases in which the assignment does not match its original taxon will be carefully inspected to assess whether it was produced by an error in the assignment, or if it could be a genuine case of HGT. The results are shown in Table 1 for a set of 1000 sequences, and are classified into three different taxonomic ranks (class, order and family).

**Table 1:** Benchmarking of the taxonomic assignment method for 1000 random sequences taken from complete genomes.

***Tax rank***	***ORFs***	***Predictions***	***Same***	***Different***	***Contigs***	***Predictions***	***Same***	***Different***
class	3264	2281	2271	10	996	694	692	2
order	3264	2099	2072	27	996	638	629	9
family	3264	1833	1791	42	996	553	539	14

Assignments were done at three different taxonomic depths (class, order and family). The table shows the number of predictions carried out, and how many of these match the original taxon, both for ORFs and contigs. The homology searches for four contigs did not provide any hits and were, therefore, not considered

The results indicate that the method is highly accurate at these taxonomic ranks. Precision (how well the method assigns taxa for the ORFs) was 99.5, 98.6 and 97.7% for class, order and family ranks, respectively. Recall (how many ORFs can be assigned) was also acceptable (69.3, 64.7 and 55.5%). By inspecting the 10 assignments that do not match their original taxon for the class rank, we found that 6 could correspond to real instances of HGT (see Table 2): two ORFs in a contig of *Treponema denticola *which, according to their phylogenies, are monophyletic with Clostridia; an ORF in *Streptococcus pyogenes*, that is closely related to the neighbouring taxa of Bacillales, is flanked by a transposase, and shows a rather different DNA compositional profile as compared to the rest of its contig; another ORF in a contig of *Archaeoglubus fulgidus *that finds very close homologues only with the Pyrococcus clade; and two ORFs in contigs from *Neisseria gonorrhoeae *and *Listeria innocua*, which show an atypical DNA composition and are predicted to be involved in HGT-related functions (transduction and transposition).

**Table 2:** ORFs of the random data set that were assigned to a different taxon than their original one (taxonomic rank: class)

***Species***	***Original taxon***	***Assigned taxon***	***Putative function***	***Identity***
*Treponema denticola*	Bacteria; Spirochaetes; Spirochaetales	Bacteria; Firmicutes; Clostridia	Sodium-dependent transporter	69%
			Tryptophanase	75%
*Streptococcus pyogenes*	Bacteria; Firmicutes; Lactobacillales	Bacteria; Firmicutes; Bacillales	Hypothetical protein	56%
*Neisseria gonorrhoeae*	Bacteria; Proteobacteria; Betaproteobacteria	Bacteria; Proteobacteria; Gammaproteobacteria	Cro-like protein	57%
*Listeria innocua*	Bacteria; Firmicutes; Bacillales	Bacteria; Firmicutes; Lactobacillales	Transposase	100%
*Archaeoglobus fulgidus*	Archaea; Euryarchaeota; Archaeoglobi	Archaea; Euryarchaeota; Thermococci	Hypothetical protein	87%
*Prochlorococcus marinus*	Bacteria; Cyanobacteria; Prochlorales	Bacteria; Cyanobacteria; Chroococcales	DNA polymerase	73%
			thymidylate kinase	68%
			P-type ATPase	72%
*Prochlorococcus marinus*	Bacteria; Cyanobacteria; Prochlorales	Bacteria; Cyanobacteria; Chroococcales	protoheme farnesyltransferase	87%

Assignments were done at three different taxonomic depths (class, order and family). The table shows the number of predictions carried out, and how many of these match the original taxon, both for ORFs and contigs. The homology searches for four contigs did not provide any hits and were, therefore, not considered

HGT is less likely in the four remaining cases. They belong to the cyanobacteria *Prochlorococcus marinus *(cyanobacteria/prochlorales), and are assigned to their closest taxon (cyanobacteria/chroococcales). As we will describe carefully in the next section, these assignments are probably due to database incompleteness: no homologues from the original taxon can be found, and the ORF is assigned to a different but close taxon. This occurs when the taxon is rare or poorly known, which is the case for Prochlorales.

Three contigs will be proposed to belong to a taxon that differs to the original, since all its ORFs have been assigned to it: the two contigs of *Prochlorococcus marinus*, and that of *Treponema denticola*. Since the method detects HGT only when a transition is present (corresponding to short blocks of transferred genes or to the extremes of longer transferred blocks), a possible transfer will not be suspected for these contigs.

Therefore, the method will detect just three putative HGT events in the contigs of *Streptococcus pyogenes, Neisseria gonorrhoeae *and *Listeria innocua*, since the rest of the ORFs in these contigs have been correctly classified. All of them are very likely to be the product of HGT; two of them contain zones of atypical composition, and one corresponds to a transposase.

#### Database incompleteness and rare taxa

In the previous section we have shown an example of the effect that incomplete knowledge of the universe of prokaryotes can have on the performance of this method. To further investigate the influence of this factor, we have performed two different tests: a deletion experiment, in which a percentage of the database is removed to simulate a reduction in our current knowledge, and a test of assignment involving sequences from rare taxa, which probably represent the most serious challenge to the method.

In the deletion experiment, we randomly removed a percentage of the entries of the non-redundant database used for homology searches, and then performed the full procedure of taxonomic assignment for the data set of random sequences from complete genomes and an "order" taxonomic division. The results are shown in Additional file 6, and indicate that precision is not greatly affected until a large fraction of the database has been deleted. It is necessary to delete more than 80% of the database to lose just 10% precision, while almost 90% of correct assignments remain. The deletion mainly affects recall, which decreases slightly until 60% of the database has been removed, when it drops rather rapidly. This is probably related to the taxonomic distribution of the entries in the database. Removing a few entries only has a slight effect on the best known groups (also the most represented groups in the data set used for the test) since it is likely that closely related sequences can still be found. This result also indicates that the phylogenetic method is expected to improve over time since the completion of the databases will increase the number of predictions that can be made.

It is important to test the performance of the method when working with sequences of poorly known taxa since many metagenomes could be enriched in these rare taxa. There are three possible outcomes, which from best to worst are: the method can produce an assignment only when the case is clear and leaves it unassigned otherwise; or it can fail systematically, by always assigning ORFs to the same close taxa; or it can fail completely and assign ORFs to different and incorrect taxa.

The results of the assignment for a data set of rare taxa (see Methods) are shown in Additional file 2. Once more, they indicate a high precision of the assignments. Dehalococcoidetes is the taxon that achieves the best results: 63% of the ORFs and 85% of the contigs were assigned with almost 100% precision. Only two ORFs were assigned to a different taxon (Firmicutes), a likely candidate for HGT since compositional analysis indicates that the contig presents a marked transition. The good results for this taxon are due to the availability of two closely related complete genomes, and they illustrate the situation well in which having a single complete genome can be of great help in the analysis of environmental sequences.

For Planctomycetacia, the method classified just 10% of the ORFs and contigs, but did so with utmost precision (100%). In this case, a prediction was made only for clear cases in which reliable homologues could be found.

In the case of Methanopyri, all sequences in the database belonging to that taxon originate from a single organism:*Methanopyrus kandleri*. Since sequences from the same species are excluded for benchmarking (see Methods), it is clear that any possible assignment will be incorrect. In this extreme case, the method produces very few results: only five ORFs (1% of the total) are assigned, all of them to other Euryarchaeota.

In summary, the results of these tests indicate that the method is very accurate in all cases and quite sensitive when enough information is available. When the original taxon is not well known, the method acts conservatively and produces very few assignments.

#### Composition of the community

**Figure 3:**
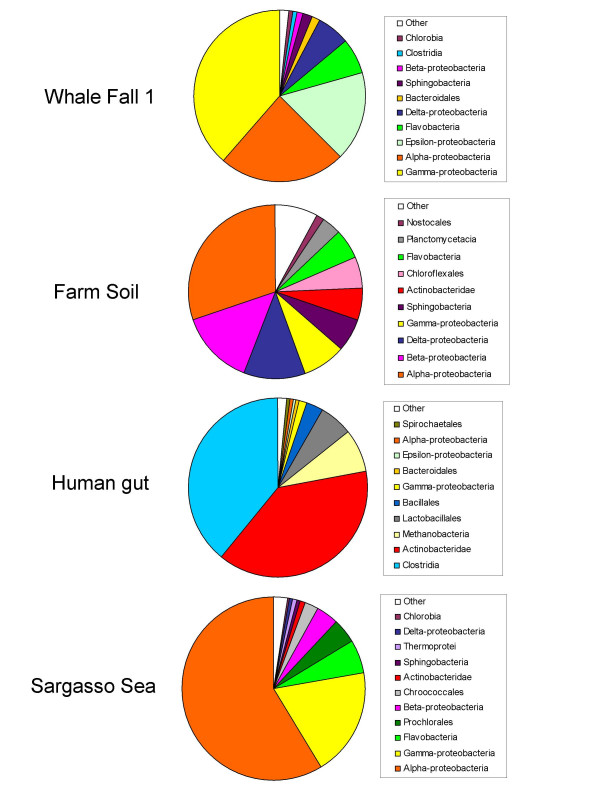
Composition of the prokaryotic communities in different metagenomes resulting from the taxonomic assignments made by the phylogenetic method.

The taxonomic assignments for real metagenomes can be used to study the composition of the different communities. The percentage of sequences assigned to different taxa for the four metagenomes under study is shown in Figure 3 and in Additional file 2. The results for all metagenomes are in good agreement with the compositions based on 16S rDNA libraries reported in the original papers, which can also be seen as an additional support to the quality of the assignments. All communities are rich and diverse, and are representative of many different taxa. A single taxon, alpha-proteobacteria, seems to be ubiquous and is the most abundant one in soil and Sargasso Sea metagenomes, but is also very abundant in the whale fall sample. In contrast, some taxa seem to be very environment-specific, such as Planctomycetales in soil, Prochlorales in sea or Lactobacilli in the gut.

In the gut and Sargasso Sea metagenomes, two taxa are clearly predominant (Clostridia and Actinobacteria in the gut, alpha- and gamma-proteobacteria in Sargasso Sea). The whale fall metagenome is dominated by proteobacterial taxa, particularly alpha-, gamma- and epsilon-proteobacteria. The soil metagenome shows the most balanced composition. No taxon dominates clearly and many taxa are represented in substantial amounts, which is in agreement with the highly diverse distribution found in 16S rDNA sequences [33]. The farm soil metagenome also has the largest amount of sequence for which no homologues can be found; that is, the highest number of putative orphans (Additional file 2). In contrast, a recent study of 16S samples from different environments concludes that soil samples are less diverse, which we believe to be the result of the biases in the method used for estimating diversity [43]. Our study confirms the phylogenetic richness and diversity of the prokaryotic soil community.

#### Quantifying possible HGT events in metagenomes by taxonomic assignment

As mentioned above, a possible HGT event in a metagenomic contig is predicted whenever it contains ORFs assigned to different taxa. We analysed the number of such cases found in the four metagenomes. As these metagenomes come from very different environments and conditions, their communities could reflect rather different lifestyles. We wanted to test whether there were significant differences in the relative amount of putative HGT that can be detected in these metagenomes by this procedure.

The results (Table 3) show that the number of detected HGT events was low in all cases, and that it matched well with what was found when analysing HGT in protein families using other methods based on phylogenies [12,14]. Obviously, the putative transfers within taxa must be much more abundant; however, they cannot be detected by this method. A higher number of events was detected for the gut and Sargasso Sea ones. Interestingly, one of the marine metagenomes (whale fall) almost doubled the quantity of HGT found for the other (Sargasso Sea).

**Table 3:** Number and percentage of contigs of the metagenomes under study containing transitions in their taxonomic assignments.

**metagenome**	**Probable HGT events**
wfall1	80/5269 **(1.5%)**
gut	37/3559 **(1.0%)**
soil	95/6481 **(1.4%)**
Sargasso	119/15552 **(0.8%)**

Only contigs with two ORFs or more were considered. The total number of contigs considered is also shown.

We can also study the taxa involved in the transferences, although it is not possible to know the direction of the transference in the small metagenomic sequences, that is, distinguishing donor from the receptor. The most frequently involved taxa in putative transferences are presented in Additional file 2. As expected, the involved taxa are either those most abundant in the metagenome, or those for which evidence of frequent transferences exist [13]. An interesting exception can be found in the Sargasso Sea metagenome, where many transfers involving a flavobacteria and either gamma- or alpha-proteobacteria are detected, a trend that has not been found before.

### Compositional method

#### Benchmarking

The objective is to provide an estimation of the accuracy of the compositional method to detect changes in DNA composition that could correspond to HGT events. The method has been tested on artificial contigs extracted from the genome of *E. coli*, and the results of different HGT predictions in that genome [28] have been used to assess the goodness of the results (see Methods). The results can be seen in Table 4 and in Additional file 2.

**Table 4:** Prediction of compositional transitions.

Cluster	-/- (TN)	-/+ (FP)	+/- (FN)	+/+ (TP)	Precision TP/TP+FP	Recall TP/TP+FN	Specificity TN/TN+FP	Accuracy TP+TN/all
0.7	1899	651	546	902	58.0%	62.3%	74.5%	70.1%
0.65	2127	423	666	782	64.9%	54.0%	83.4%	72.8%
0.6	2255	295	772	676	69.6%	46.7%	88.4%	73.3%
0.55	2346	204	893	555	73.1%	38.3%	92.0%	72.6%
0.5	2427	123	976	472	79.3%	32.6%	95.2%	72.5%
0.4	2495	55	1114	334	85.9%	23.1%	97.8%	70.8%
0.3	2522	28	1219	229	89.1%	15.8%	98.9%	68.8%

Number of true negatives, false positives, false negatives, true positives, and the measures of precision, recall, specificity and accuracy are shown. Different values of the clustering parameter have been used.

When an intermediate value of the clustering parameter is chosen (0.5), the method is able to detect approximately one third of the transitions with a high precision (80%). As expected, the method underestimates the number of compositional transitions given the lack of contextual information, and therefore that of putative HGT events. It must be remarked that the number of wrong predictions is low, and therefore this method can be used to measure the relative number of possible HGT events in different sets of sequences. It should also be noticed that according to Additional file 2, many of the false positives correspond to cases in which one of the reference methods predicts HGT, while the other does not. In the event of considering these instances as likely cases of HGT, the performance of the method will improve substantially with regard to precision and recall. Precision will reach values of 98%, 87% and 81% for clustering parameters 0.3, 0.6 and 0.7, respectively.

#### Quantifying HGT in metagenomes by DNA composition

We have used the compositional method to analyse the number of likely HGT events that can be found in the metagenomes under study. The results are shown in Table 5.

**Table 5:** Number of contigs in the four metagenomes showing compositional transitions, which could correspond to HGT events

	**Contigs**	**0.4**	**0.5**	**0.6**
**Whale fall**	3739	153 **(4.1%)**	301 **(8.0%)**	558 **(14.9%)**
**Human gut**	2831	72 **(2.5%)**	111 **(3.9%)**	185 **(6.5%)**
**Farm soil**	5077	125 **(2.5%)**	211 **(4.2%)**	387 **(7.6%)**
**Sargasso Sea**	10654	121 **(1.1%)**	234 **(2.2%)**	415 **(3.9%)**

Only contigs with two ORFs or more that are longer than 250 base pairs were considered. Three different values of the clustering parameter were used, but the trend of the results remains the same.

Three different values of the clustering parameter have been used. Lower values show low recall and high precision, while higher values balance both measures. The range of HGT percentages found (between 2% and 8%, without considering extreme values) is consistent with those reported for complete genomes [28]. The trend remains the same for all values of the clustering parameter: the highest percentage of compositional transitions was found for the whale fall metagenome, followed by the human gut and farm soil metagenomes, which revealed very similar percentages; then finally, Sargasso Sea, which is well below the others and shows the lowest number of compositional transitions.

**Figure 4:**
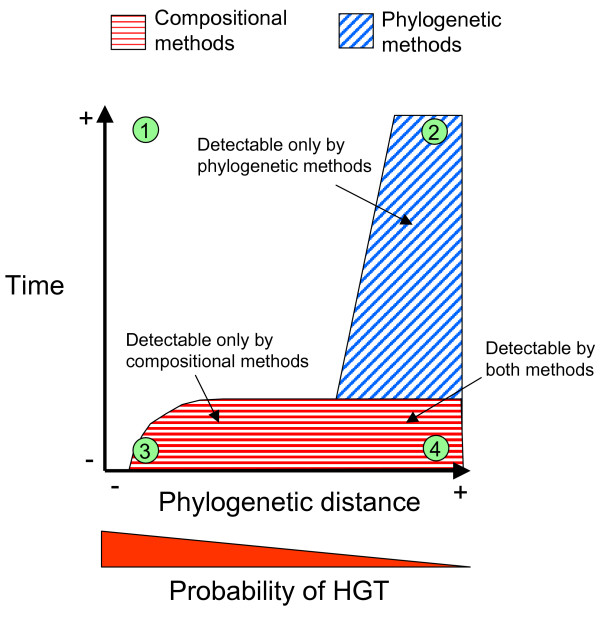
**Probability of detecting HGT for compositional and phylogenetic methods by taking into account the time since the event and the phylogenetic distance between donor and receptor.** The shaded surfaces are these combinations of time and the phylogenetic distance for which a HGT can be detected by each method. As composition ameliorates faster than phylogenies, old events can only be detected using phylogenetic methods, and only when they involve distant taxa. Compositional methods usually detect more events since HGT seems to occur preferentially between close taxa, where the resolution of phylogenetic methods is low. A putative event can be identified by both methods when both surfaces overlap; that is, when it involves relatively distant taxa and happened recently. The exact shape of the surfaces indicating the capacity of the methods is as yet unknown. The green points indicate examples of different HGT events: 1) Old events between closely related taxa. We will observe normal composition (by amelioration), and congruent trees (not enough resolution since species are close). The event will be undetectable by both methods; 2) Old events between distant taxa. Normal composition, incongruent trees. Detectable by phylogenetic methods; 3) Recent events between close taxa. Atypical composition, congruent trees. Detectable by compositional methods; 4) Recent events between distant taxa. Atypical composition, incongruent trees. Detectable by both methods.

Although both methods presented herein are apparently quite accurate in the detection of putative HGT events, most of their predictions are not coincident, and less than 5% of the predictions are shared by both methods. This can be explained if we consider that the HGT events detected by DNA composition are often undetectable by taxonomic assignment because their phylogenies are noisy or inconclusive (for instance, see Figure 1 or Additional file 7). This can indicate that these genes have a complex history of multiple acquisitions and losses in different genomes. Notice that, in the context of a complete genome, these events would easily be detected by phylogenetic methods since we could see that the evolutionary history of the ORF is rather different to other ORFs in the genome. In this scenario, compositional and phylogenetic methods would agree in the prediction, as already proposed [30]. In addition, those events detected by phylogenies are often not detected by composition. This is likely because these are old events involving distant taxa that have already been ameliorated (Figure 4).

Nevertheless, the results from both methods are consistent and show a similar trend: the whale fall metagenome presents the highest number of detectable HGT events. The soil metagenome comes second, closely followed by the human gut metagenome. Finally, and perhaps unexpectedly, the Sargasso Sea metagenome is the one in which less HGT events can be identified by both methods. This low amount of HGT found for the Sargasso Sea metagenome is remarkable since it has been proposed that the high quantities of bacteriophages found in sea waters can promote HGT significantly [44-46].

## Conclusion

The task of detecting HGT in metagenomic sequences is difficult. The available methods are designed to work with complete genomes (or at least with a considerable amount of sequence), and it is assumed that one knows the species from which the sequences are taken: phylogenetic methods attempt to identify deviations with regard to the expected phylogeny, while compositional methods detect differences in the compositional profile of each ORF as compared to that of the complete genome. These methods are not applicable to metagenomic sequences. Metagenomes are usually composed of a complex mixture of short sequences belonging to many different species.

Not knowing the source species for each sequence in the metagenome is the most serious hurdle for all metagenomic studies. One of the most relevant advances in metagenome analysis will be to solve the problem of binning, that is, the assignment of metagenomic sequences to source species or taxa. Although some attempts have been made by compositional methods to address this task [47-49], the problem remains unresolved. As it stands, more complete genomes would be required for training, as would longer sequences than those currently supplied by metagenomic projects [50]. Nevertheless, it is likely that the length of the sequences obtained in these projects will not change very much in the near future since metagenomics is tightly linked to the use of fast and cheap pyrosequencing machinery [51], which currently obtains short readings and contigs (although considerably efforts are being made to improve read lengths). Therefore, either more powerful methods or different approaches will be needed to address the task of assigning sequences to species or taxa. We have presented herein the usage of a highly precise method, based on sequence homology, which can provide an interesting alternative for complementing compositional approaches. The advantages of methods based on sequence homology and phylogenies are that they are unaffected by the short length of the contigs, they can work with partial sequences, and can even classify relatively unknown taxa with high accuracy. It is also possible to take advantage of the presence of several ORFs in the contig to support the assignments, thus increasing precision and, especially, sensitivity.

It is very likely that both methods presented herein significantly underestimate the number of HGT events. Compositional methods can only detect recent transferences before amelioration erases the distinctive signatures of the sequences. When dealing with metagenomic sequences, the situation is even more challenging since the analysis must only be done for a very short segment of the sequence, and we lack the contextual information provided by the rest of the genome. On the other hand, phylogenetic methods usually rely on distinguishing incongruent phylogenies, identifying the branches of a tree that are in atypical positions with respect to a reference. Once again, this is a difficulty for metagenomic sequences because the species source for the sequence is unknown. Therefore, it is not possible to assess its position in the tree. We rely on assigning the most probable taxon for each ORF to distinguish instances of neighbouring sequences of different likely origins. The main disadvantage of this procedure is that it has a limited sensitivity. It is very difficult and risky to perform assignments at deep taxonomic ranks, and it is probably impossible to do so at the species level. Therefore, we will only detect events involving distant taxa, and will overlook those that happen between closely related species or taxa, which are probably the most abundant.

The results presented in this work must be taken as an indication of possible trends for HGT events in prokaryotic samples from diverse environments. However, it must be taken into account that many different factors, some yet unknown, can influence the prevalence of HGT in a community. For instance, we do not know how the composition of the community shapes the likelihood of HGT events. HGT could be expected to be widespread in a very diverse community in which many different genes and subsystems are present, and transferences could greatly accelerate evolution and adaptation to environmental challenges [52]. On the other hand, HGT might be easier in a community composed of closely related species for which the barriers to the transference can be more easily surmounted [17]. Furthermore, some species seem to be more prone to HGT events than others [53-55]. The prevalence of HGT events is certainly influenced by both environmental characteristics and the composition of the community, and as yet, we do not know the balance between both factors. Furthermore, we must realize that we are observing putatively transferred genes in the genomes of the species present in these communities. Obviously, these species are present in other communities and environments, and the putative HGT events may have occurred in any of these. For instance, we have found transferences between different archaea in the human gut metagenome (Additional file 2). The only archaea that has been found in measurable amounts in the human gut is *Methanobrevibacter smithii*. This indicates that these putative transfers could have happened in some other environment in which *M. smithii *can co-exist with other archaea, for example, sewage waters. A similar circumstance has been found in another study of bacteria from the human intestine [7].

This study attempts to provide the first methodological step for the study of the dynamics of HGT in prokaryotic communities. Further studies should deal with the influence that community composition and environmental factors have on HGT rates by addressing questions such as: Is HGT more widespread in more diverse communities? Or is it favoured in cases where the community is dominated by a few closely related taxa? To what extent do environmental challenges affect the rate of gene transfer? Is there a correlation between the amount of transferences and the presence of bacteriophages?

The answer to these questions will shed light on the true role of HGT in adaptation and innovation, and will greatly improve our understanding of the dynamics of natural prokaryotic communities.

## Authors' contributions

JT conceived the study and performed the analyses. AM participated in the coordination, and helped to draft the manuscript. Both authors participated in the discussion of the biological implications of the results, and they read and approved the final manuscript.

## Supplementary Material

Additional file 1 Metagenomic data. This figure shows the contig length and number of ORFs per contig in different metagenomes. Click here for file

Additional file 2 Supplementary tables. Supplementary table 1: Species and taxonomic classes for the contigs in the random dataset; Supplementary table 2: Results of the phylogenetic assignment of 100 sequences from rare taxa; Supplementary table 3: Number of assignments (class taxonomic rank) for the contigs of different metagenomes. Supplementary table 4: Length of sequence analyzed, length of sequence with homologues, and percentage of sequence for which no homologues could be found Supplementary table 5: Taxa involved in possible HGT events (phylogenetic method). Supplementary table 6: Number of contigs proposed to contain a compositional transition (compositional method) Click here for file

Additional file 3 ORF determination. Blastx run of a metagenomic contig, indicated by the red bar on the top. Eleven hits (homologue proteins) have been found, shown in black. Grey segments in the hits indicate the part of the homologue protein that has not been found. In the merging step, protein hits 1–4 are considered homologues since they are in the same position and frame containing the full length of the protein hit. Therefore they are merged in a single hit. Hits 7–10 are also in the same position and frame, but the alignment covers less than 50% of the protein hit because it is truncated by the end of the contig. In this case, as the other extreme of the protein has been found, the hit is considered valid, and homologues merge as before. Protein hit 5 is in the same frame as hits 1–4, but is much shorter. Therefore, it does not merge with hits 1–4, and is removed in the filtering step where all short hits overlapping with others in the position and frame are removed. Note that protein hit 6 overlaps slightly with hits 1–5, but it is considered a different ORF since they overlap by less than 50% of the length of both proteins. Protein 11 is removed in the filtering step since it covers less than 50% of the protein hit and is not truncated by the extremes of the contig. Click here for file

Additional file 4 Example of the phylogenetic method. Three examples of the procedure for the taxonomic assignment by the phylogenetic method. Phylogenetic trees have been created with the homologues found for three different metagenomic ORFs. Homologues are coloured according to their taxonomic affiliation. The position of the query metagenomic ORF is signalled by the black arrows in the trees. The tables at the bottom of the trees show the sorted list of the homologues and their distances to the query ORF. A) The query ORF is monophyletic with the Epsilon-proteobacteria taxon. Therefore, the lowest distance scores are those for the homologues belonging to that taxon, and the query ORF is automatically assigned to it. B) The ORF is closely related to alpha-proteobacteria, but there are some homologues belonging to that taxa that are distantly related. Nevertheless, the average distance score for alpha-proteobacteria is more than 15% lower than the average distance for any other taxon, thus allowing its assignment to it. C) In this case, neither the ORF is monophyletic with any taxon, nor the average distance scores allow the assignment, and the query ORF remains unassigned. Click here for file

Additional file 5 Example of the compositional method. Compositional analysis of several contigs. The upper left panel corresponds to the usual pattern for a compositional homogeneous contig. The other three panels show compositional transitions. Click here for file

Additional file 6 Influence of database size. Effect of the deletion of a variable percentage of the database used in the performance of taxonomic assignments. The ordinate axis shows the percentage of entries deleted from the database, while the abscissa axis shows the percentage of precision (TP/TP+FP) and sensitivity (TP/TP+FN) for the assignment of the ORFs. Click here for file

Additional file 7 Full analysis of a single contig. Analysis of contig AAFY01000115 from the whale fall metagenome. Homology searches indicate that the contig contains three ORFs. A: The compositional method identifies a transition in the contig. The first two ORFs show a similar composition, but the third differs. In addition, the second ORF is a transposase, which supports the idea of a probable HGT. B: Taxonomic assignment provides a result for the two first ORFs (alpha-proteobacteria), but not for the third. The third ORF finds only one distant homologue (27% identity) with a gamma-proteobacteria (*Acinetobacter sp*.), and therefore an assignment cannot be made. As a result, this contig is recognised as a probable HGT only by the compositional method. Click here for file
