# Supplementary figures and images for: Estimating the extent of horizontal gene transfer in metagenomic sequences
Source: BMC Genomics. 2008 Mar 24;9:136. doi: 10.1186/1471-2164-9-136 (PMC2324111; doi:10.1186/1471-2164-9-136)

## Slide 1
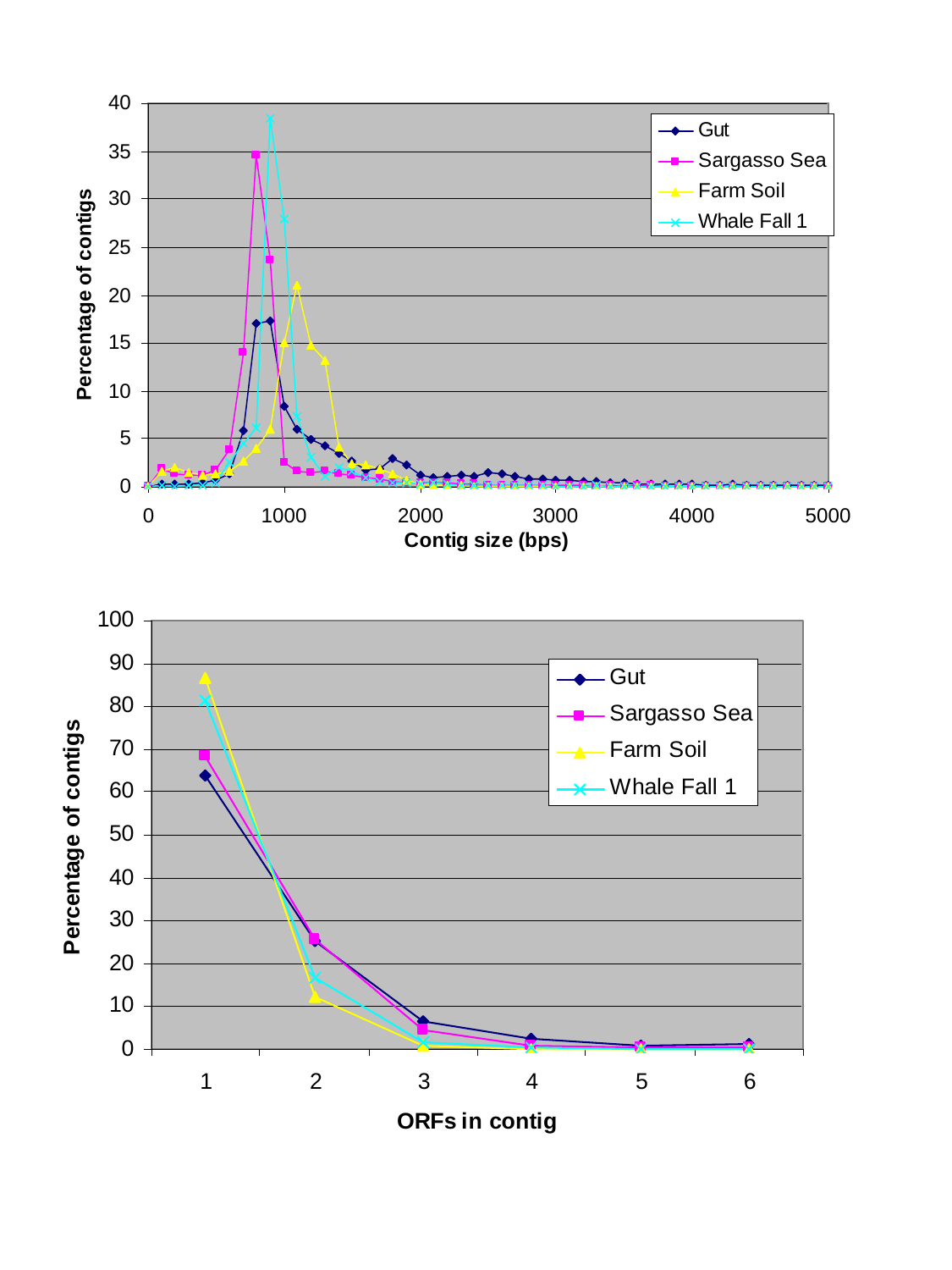

Supplement: Additional file 1 — Metagenomic data. This figure shows the contig length and number of ORFs per contig in different metagenomes. [file 1471-2164-9-136-S1.ppt]

## Slide 1
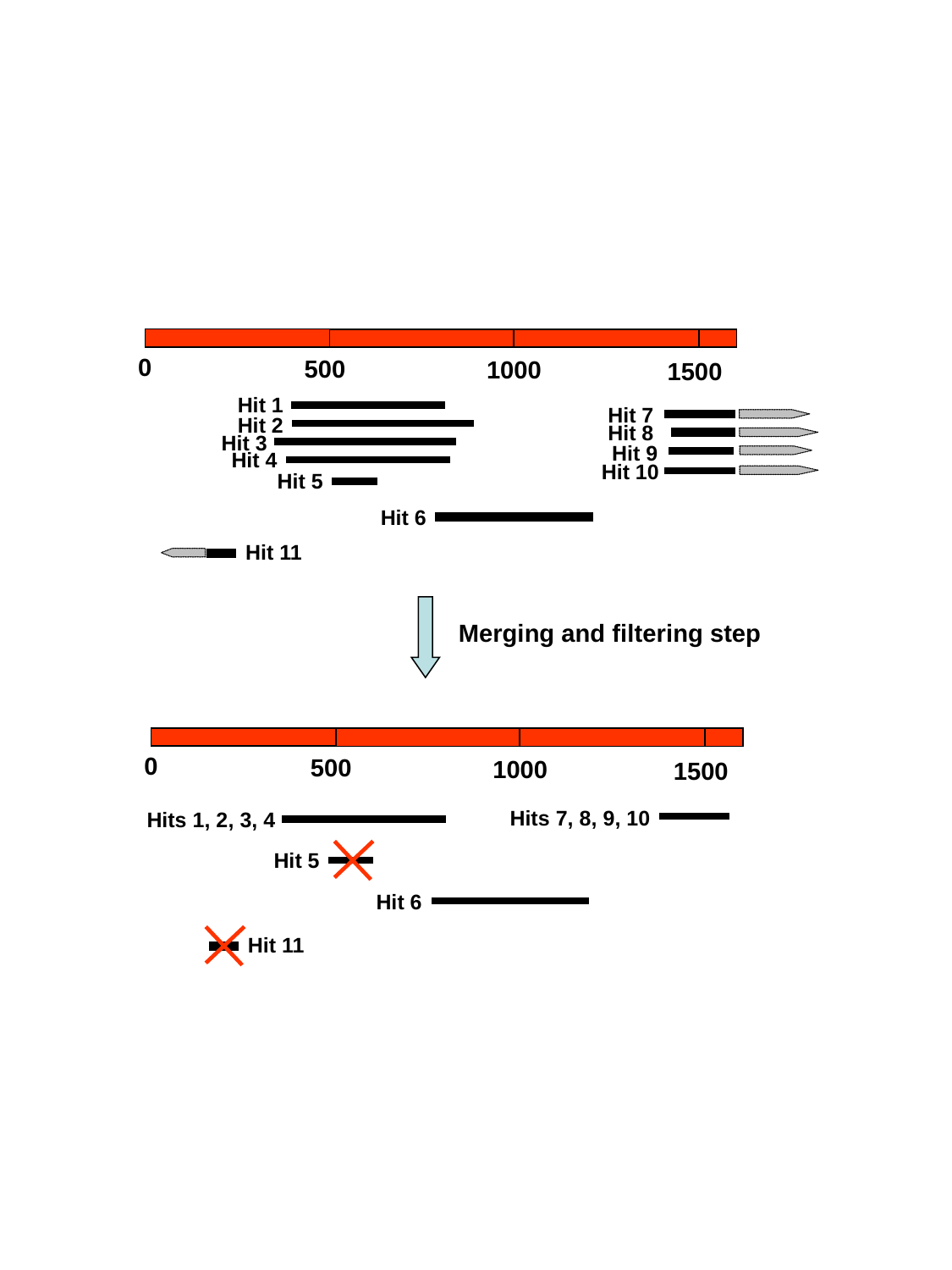

0
500
1000
1500
Hit 1
Hit 7
Hit 2
Hit 8
Hit 3
Hit 9
Hit 4
Hit 10
Hit 5
Hit 6
Hit 11
Merging and filtering step
0
500
1000
1500
Hits 7, 8, 9, 10
Hits 1, 2, 3, 4
Hit 5
Hit 6
Hit 11

Supplement: Additional file 3 — ORF determination. Blastx run of a metagenomic contig, indicated by the red bar on the top. Eleven hits (homologue proteins) have been found, shown in black. Grey segments in the hits indicate the part of the homologue protein that has not been found. In the merging step, protein hits 1–4 are considered homologues since they are in the same position and frame containing the full length of the protein hit. Therefore they are merged in a single hit. Hits 7–10 are also in the same position and frame, but the alignment covers less than 50% of the protein hit because it is truncated by the end of the contig. In this case, as the other extreme of the protein has been found, the hit is considered valid, and homologues merge as before. Protein hit 5 is in the same frame as hits 1–4, but is much shorter. Therefore, it does not merge with hits 1–4, and is removed in the filtering step where all short hits overlapping with others in the position and frame are removed. Note that protein hit 6 overlaps slightly with hits 1–5, but it is considered a different ORF since they overlap by less than 50% of the length of both proteins. Protein 11 is removed in the filtering step since it covers less than 50% of the protein hit and is not truncated by the extremes of the contig. [file 1471-2164-9-136-S3.ppt]

## Slide 1
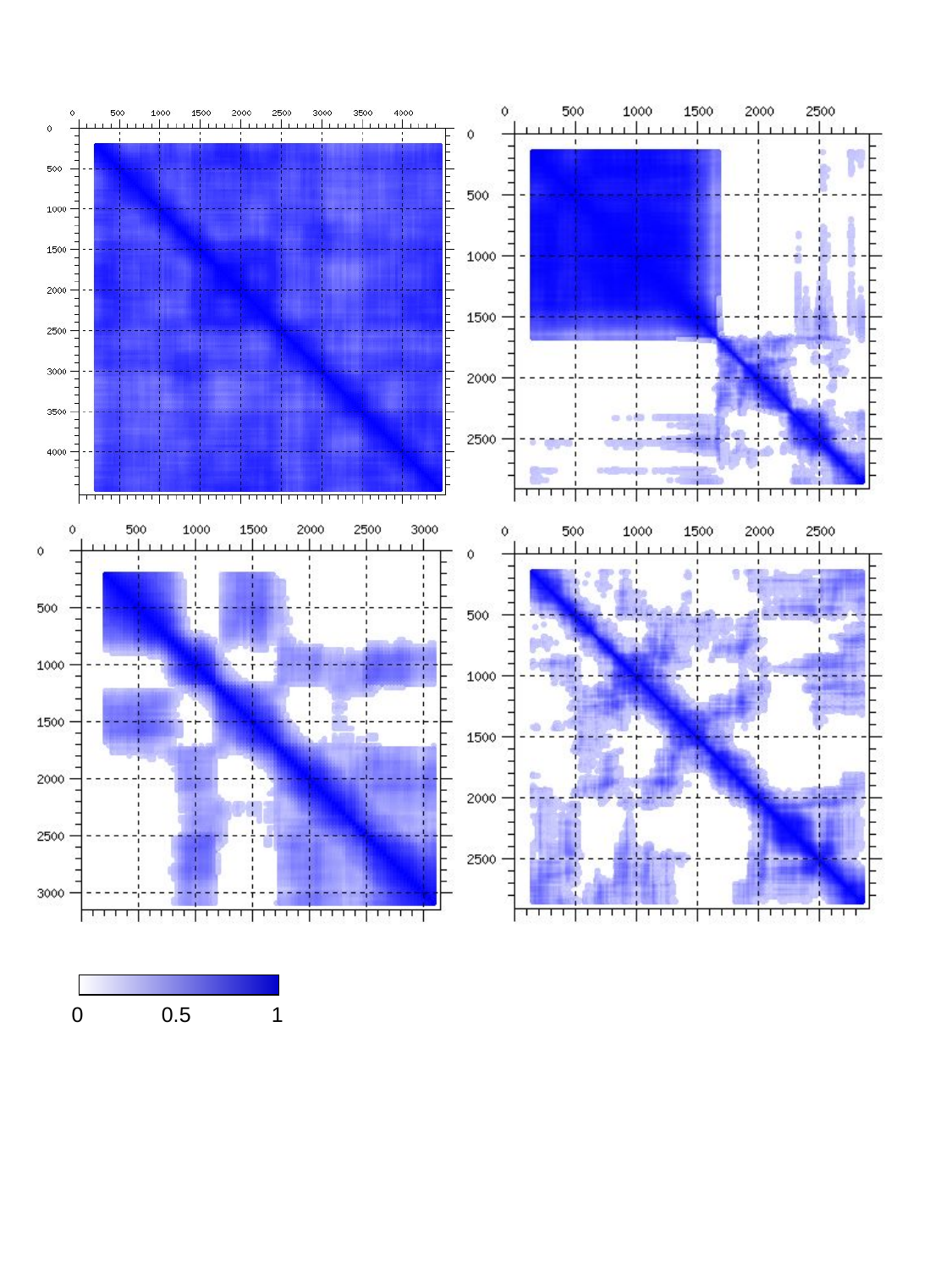

0
0.5
1

Supplement: Additional file 5 — Example of the compositional method. Compositional analysis of several contigs. The upper left panel corresponds to the usual pattern for a compositional homogeneous contig. The other three panels show compositional transitions. [file 1471-2164-9-136-S5.ppt]

## Slide 1
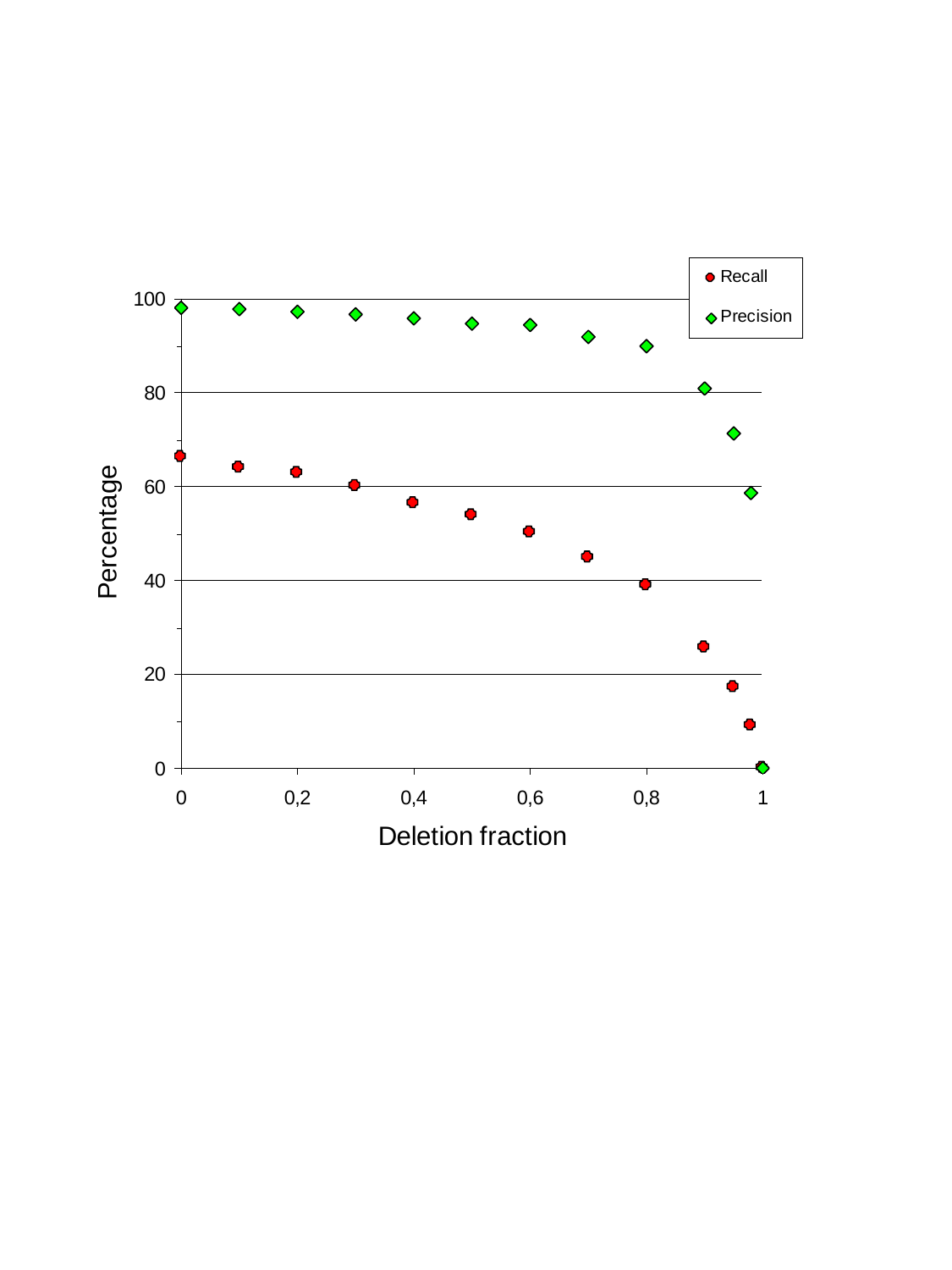

Supplement: Additional file 6 — Influence of database size. Effect of the deletion of a variable percentage of the database used in the performance of taxonomic assignments. The ordinate axis shows the percentage of entries deleted from the database, while the abscissa axis shows the percentage of precision (TP/TP+FP) and sensitivity (TP/TP+FN) for the assignment of the ORFs. [file 1471-2164-9-136-S6.ppt]
