# Supplementary material for: Estimating the extent of horizontal gene transfer in metagenomic sequences
Source: BMC Genomics. 2008 Mar 24;9:136. doi: 10.1186/1471-2164-9-136 (PMC2324111; doi:10.1186/1471-2164-9-136)
Supplement: Additional file 2 — Supplementary tables. Supplementary table 1: Species and taxonomic classes for the contigs in the random dataset; Supplementary table 2: Results of the phylogenetic assignment of 100 sequences from rare taxa; Supplementary table 3: Number of assignments (class taxonomic rank) for the contigs of different metagenomes. Supplementary table 4: Length of sequence analyzed, length of sequence with homologues, and percentage of sequence for which no homologues could be found Supplementary table 5: Taxa involved in possible HGT events (phylogenetic method). Supplementary table 6: Number of contigs proposed to contain a compositional transition (compositional method) [file 1471-2164-9-136-S2.doc]

**Supplementary table 1**

| **Species** | **Class** | **seqs** |
| --- | --- | --- |
| *Syntrophomonas wolfei subsp wolfei str Goettingen* | Clostridia | 9 |
| *Bacillus anthracis str Ames Ancestor* | Bacillales | 7 |
| *Escherichia coli K12* | Gammaproteobacteria | 7 |
| *Campylobacter jejuni RM1221* | Epsilonproteobacteria | 7 |
| *Pseudoalteromonas haloplanktis TAC125* | Gammaproteobacteria | 7 |
| *Rhodopseudomonas palustris BisB5* | Alphaproteobacteria | 7 |
| *Mesorhizobium loti MAFF303099* | Alphaproteobacteria | 7 |
| *Streptococcus pyogenes MGAS10394* | Lactobacillales | 6 |
| *Methanospirillum hungatei JF-1* | Methanomicrobia | 6 |
| *Anaplasma phagocytophilum HZ* | Alphaproteobacteria | 6 |
| *Pyrobaculum aerophilum str IM2* | Thermoprotei | 6 |
| *Anabaena variabilis ATCC 29413* | Nostocales | 6 |
| *Cytophaga hutchinsonii ATCC 33406* | Sphingobacteria | 6 |
| *Streptococcus pyogenes MGAS315* | Lactobacillales | 6 |
| *Nitrosomonas eutropha C71* | Betaproteobacteria | 6 |
| *Clostridium acetobutylicum ATCC 824* | Clostridia | 6 |
| *Pseudomonas syringae pv phaseolicola 1448A* | Gammaproteobacteria | 6 |
| *Mycoplasma synoviae 53* | Mollicutes | 6 |
| *Arthrobacter sp FB24* | Actinobacteridae | 6 |
| *Rhodopseudomonas palustris BisA53* | Alphaproteobacteria | 6 |
| *Archaeoglobus fulgidus DSM 4304* | Archaeoglobi | 5 |
| *Borrelia afzelii PKo* | Spirochaetales | 5 |
| *Fusobacterium nucleatum subsp nucleatum ATCC 25586* | Fusobacterales | 5 |
| *Photorhabdus luminescens subsp laumondii TTO1* | Gammaproteobacteria | 5 |
| *Synechococcus elongatus PCC 6301* | Chroococcales | 5 |
| *Caulobacter crescentus CB15* | Alphaproteobacteria | 5 |
| *Carboxydothermus hydrogenoformans Z-2901* | Clostridia | 5 |
| *Geobacter sulfurreducens PCA* | Deltaproteobacteria | 5 |
| *Aeromonas hydrophila subsp hydrophila ATCC 7966* | Gammaproteobacteria | 5 |
| *Agrobacterium tumefaciens str C58* | Alphaproteobacteria | 5 |
| *Buchnera aphidicola str Bp Baizongia pistaciae* | Gammaproteobacteria | 5 |
| *Vibrio parahaemolyticus RIMD 2210633* | Gammaproteobacteria | 5 |
| *Tropheryma whipplei str Twist* | Actinobacteridae | 5 |
| *Neisseria gonorrhoeae FA 1090* | Betaproteobacteria | 5 |
| *Trichodesmium erythraeum IMS101* | Oscillatoriales | 5 |
| *Lactobacillus casei ATCC 334* | Lactobacillales | 5 |
| *Deinococcus geothermalis DSM 11300* | Deinococci | 5 |
| *Lactobacillus delbrueckii subsp bulgaricus ATCC 11842* | Lactobacillales | 5 |
| *Streptococcus mutans UA159* | Lactobacillales | 5 |
| *Bacillus subtilis subsp subtilis str 168* | Bacillales | 5 |
| *Streptococcus pneumoniae R6* | Lactobacillales | 5 |
| *Wolbachia endosymbiont strain TRS of Brugia malayi* | Alphaproteobacteria | 5 |
| *Campylobacter jejuni subsp jejuni NCTC 11168* | Epsilonproteobacteria | 5 |
| *Alkalilimnicola ehrlichei MLHE-1* | Gammaproteobacteria | 5 |
| *Ralstonia eutropha JMP134* | Betaproteobacteria | 4 |
| *Synechococcus sp CC9902* | Chroococcales | 4 |
| *Leptospira interrogans serovar Lai str 56601* | Spirochaetales | 4 |
| *Azoarcus sp EbN1* | Betaproteobacteria | 4 |
| *Francisella tularensis subsp holarctica* | Gammaproteobacteria | 4 |
| *Mycobacterium avium subsp paratuberculosis K-10* | Actinobacteridae | 4 |
| *Pediococcus pentosaceus ATCC 25745* | Lactobacillales | 4 |
| *Rickettsia typhi str Wilmington* | Alphaproteobacteria | 4 |
| *Rhodopirellula baltica SH 1* | Planctomycetacia | 4 |
| *Listeria welshimeri serovar 6b str SLCC5334* | Bacillales | 4 |
| *Psychrobacter cryohalolentis K5* | Gammaproteobacteria | 4 |
| *Aquifex aeolicus VF5* | Aquificales | 4 |
| *Staphylococcus aureus subsp aureus MRSA252* | Bacillales | 4 |
| *Buchnera aphidicola str Sg Schizaphis graminum* | Gammaproteobacteria | 4 |
| *Desulfotalea psychrophila LSv54* | Deltaproteobacteria | 4 |
| *Synechocystis sp PCC 6803* | Chroococcales | 4 |
| *Rickettsia felis URRWXCal2* | Alphaproteobacteria | 4 |
| *Escherichia coli W3110* | Gammaproteobacteria | 4 |
| *Haloquadratum walsbyi DSM 16790* | Halobacteria | 4 |
| *Brucella suis 1330* | Alphaproteobacteria | 4 |
| *Lactobacillus gasseri ATCC 33323* | Lactobacillales | 4 |
| *Onion yellows phytoplasma OY-M* | Mollicutes | 4 |
| *Mycoplasma mobile 163K* | Mollicutes | 4 |
| *Haemophilus influenzae Rd KW20* | Gammaproteobacteria | 4 |
| *Staphylococcus haemolyticus JCSC1435* | Bacillales | 4 |
| *Xanthomonas campestris pv campestris str 8004* | Gammaproteobacteria | 4 |
| *Myxococcus xanthus DK 1622* | Deltaproteobacteria | 4 |
| *Sulfolobus acidocaldarius DSM 639* | Thermoprotei | 4 |
| *Pasteurella multocida subsp multocida str Pm70* | Gammaproteobacteria | 4 |
| *Chlamydophila pneumoniae J138* | Chlamydiales | 4 |
| *Ralstonia solanacearum GMI1000* | Betaproteobacteria | 4 |
| *Treponema denticola ATCC 35405* | Spirochaetales | 4 |
| *Lactobacillus johnsonii NCC 533* | Lactobacillales | 4 |
| *Candidatus Blochmannia floridanus* | Gammaproteobacteria | 4 |
| *Shewanella denitrificans OS217* | Gammaproteobacteria | 4 |
| *Mycobacterium tuberculosis CDC1551* | Actinobacteridae | 4 |
| *Methanococcus maripaludis S2* | Methanococci | 4 |
| *Ralstonia metallidurans CH34* | Betaproteobacteria | 4 |
| *Escherichia coli O157H7 EDL933* | Gammaproteobacteria | 4 |
| *Acidobacteria bacterium Ellin345* | Acidobacteriales | 4 |
| *Escherichia coli UTI89* | Gammaproteobacteria | 4 |
| *Streptococcus pyogenes MGAS6180* | Lactobacillales | 4 |
| *Pseudomonas putida KT2440* | Gammaproteobacteria | 4 |
| *Streptococcus pneumoniae TIGR4* | Lactobacillales | 4 |
| *Streptococcus pyogenes MGAS10270* | Lactobacillales | 4 |
| *Aeropyrum pernix K1* | Thermoprotei | 4 |
| *Jannaschia sp CCS1* | Alphaproteobacteria | 4 |
| *Solibacter usitatus Ellin6076* | Solibacteres | 4 |
| *Alcanivorax borkumensis SK2* | Gammaproteobacteria | 4 |
| *Rhodospirillum rubrum ATCC 11170* | Alphaproteobacteria | 4 |
| *Haemophilus ducreyi 35000HP* | Gammaproteobacteria | 3 |
| *Leuconostoc mesenteroides subsp mesenteroides ATCC 8293* | Lactobacillales | 3 |
| *Methanosarcina mazei Go1* | Methanomicrobia | 3 |
| *Synechococcus sp CC9311* | Chroococcales | 3 |
| *Streptococcus pyogenes MGAS9429* | Lactobacillales | 3 |
| *Streptococcus thermophilus CNRZ1066* | Lactobacillales | 3 |
| *Lactobacillus delbrueckii subsp bulgaricus ATCC BAA-365* | Lactobacillales | 3 |
| *Chlamydia muridarum Nigg* | Chlamydiales | 3 |
| *Bradyrhizobium japonicum USDA 110* | Alphaproteobacteria | 3 |
| *Vibrio cholerae O1 biovar eltor str N16961* | Gammaproteobacteria | 3 |
| *Rickettsia bellii RML369-C* | Alphaproteobacteria | 3 |
| *Thermobifida fusca YX* | Actinobacteridae | 3 |
| *Thermoanaerobacter tengcongensis MB4* | Clostridia | 3 |
| *Bdellovibrio bacteriovorus HD100* | Deltaproteobacteria | 3 |
| *Francisella tularensis subsp tularensis FSC 198* | Gammaproteobacteria | 3 |
| *Erythrobacter litoralis HTCC2594* | Alphaproteobacteria | 3 |
| *Syntrophobacter fumaroxidans MPOB* | Deltaproteobacteria | 3 |
| *Desulfovibrio desulfuricans G20* | Deltaproteobacteria | 3 |
| *Bacillus cereus ATCC 14579* | Bacillales | 3 |
| *Sodalis glossinidius str morsitans* | Gammaproteobacteria | 3 |
| *Mycobacterium tuberculosis H37Rv* | Actinobacteridae | 3 |
| *Mycoplasma pulmonis UAB CTIP* | Mollicutes | 3 |
| *Chlorobium chlorochromatii CaD3* | Chlorobia | 3 |
| *Haemophilus influenzae 86-028NP* | Gammaproteobacteria | 3 |
| *Bordetella bronchiseptica RB50* | Betaproteobacteria | 3 |
| *Ureaplasma parvum serovar 3 str ATCC 700970* | Mollicutes | 3 |
| *Symbiobacterium thermophilum IAM 14863* | Symbiobacterium | 3 |
| *Escherichia coli CFT073* | Gammaproteobacteria | 3 |
| *Chlamydophila pneumoniae AR39* | Chlamydiales | 3 |
| *Lactococcus lactis subsp lactis Il1403* | Lactobacillales | 3 |
| *Methanosaeta thermophila PT* | Methanomicrobia | 3 |
| *Rubrobacter xylanophilus DSM 9941* | Rubrobacteridae | 3 |
| *Leifsonia xyli subsp xyli str CTCB07* | Actinobacteridae | 3 |
| *Staphylococcus saprophyticus subsp saprophyticus ATCC 15305* | Bacillales | 3 |
| *Chromohalobacter salexigens DSM 3043* | Gammaproteobacteria | 3 |
| *Shigella boydii Sb227* | Gammaproteobacteria | 3 |
| *Candidatus Blochmannia pennsylvanicus str BPEN* | Gammaproteobacteria | 3 |
| *Prochlorococcus marinus subsp pastoris str CCMP1986* | Prochlorales | 3 |
| *Nocardia farcinica IFM 10152* | Actinobacteridae | 3 |
| *Streptococcus pneumoniae D39* | Lactobacillales | 3 |
| *Prochlorococcus marinus str MIT 9313* | Prochlorales | 3 |
| *Synechococcus sp JA-2-3Ba2-13* | Chroococcales | 3 |
| *Shewanella oneidensis MR-1* | Gammaproteobacteria | 3 |
| *Enterococcus faecalis V583* | Lactobacillales | 3 |
| *Thiomicrospira denitrificans ATCC 33889* | Epsilonproteobacteria | 3 |
| *Candidatus Pelagibacter ubique HTCC1062* | Alphaproteobacteria | 3 |
| *Thermus thermophilus HB27* | Deinococci | 3 |
| *Streptococcus pyogenes M1 GAS* | Lactobacillales | 3 |
| *Streptococcus agalactiae NEM316* | Lactobacillales | 3 |
| *Photobacterium profundum SS9* | Gammaproteobacteria | 3 |
| *Pelodictyon luteolum DSM 273* | Chlorobia | 3 |
| *Legionella pneumophila subsp pneumophila str Philadelphia 1* | Gammaproteobacteria | 3 |
| *Saccharophagus degradans 2-40* | Gammaproteobacteria | 3 |
| *Xanthomonas campestris pv vesicatoria str 85-10* | Gammaproteobacteria | 3 |
| *Yersinia pestis Antiqua* | Gammaproteobacteria | 3 |
| *Prochlorococcus marinus subsp marinus str CCMP1375* | Prochlorales | 3 |
| *Mycoplasma capricolum subsp capricolum ATCC 27343* | Mollicutes | 3 |
| *Mycoplasma gallisepticum R* | Mollicutes | 3 |
| *Idiomarina loihiensis L2TR* | Gammaproteobacteria | 3 |
| *Streptococcus thermophilus LMD-9* | Lactobacillales | 3 |
| *Nitrobacter winogradskyi Nb-255* | Alphaproteobacteria | 3 |
| *Corynebacterium efficiens YS-314* | Actinobacteridae | 3 |
| *Prochlorococcus marinus str NATL2A* | Prochlorales | 3 |
| *Listeria monocytogenes str 4b F2365* | Bacillales | 3 |
| *Clostridium tetani E88* | Clostridia | 3 |
| *Hyphomonas neptunium ATCC 15444* | Alphaproteobacteria | 3 |
| *Erwinia carotovora subsp atroseptica SCRI1043* | Gammaproteobacteria | 3 |
| *Brucella melitensis 16M* | Alphaproteobacteria | 3 |
| *Staphylococcus aureus subsp aureus MW2* | Bacillales | 3 |
| *Bartonella henselae str Houston-1* | Alphaproteobacteria | 3 |
| *Magnetospirillum magneticum AMB-1* | Alphaproteobacteria | 3 |
| *Salmonella enterica subsp enterica Paratyphi A ATCC 9150* | Gammaproteobacteria | 3 |
| *Lactobacillus salivarius subsp salivarius UCC118* | Lactobacillales | 3 |
| *Rickettsia prowazekii str Madrid E* | Alphaproteobacteria | 3 |
| *Salmonella enterica subsp enterica serovar Typhi Ty2* | Gammaproteobacteria | 3 |
| *Wigglesworthia glossinidia* | Gammaproteobacteria | 3 |
| *Clostridium perfringens ATCC 13124* | Clostridia | 3 |
| *Burkholderia cenocepacia HI2424* | Betaproteobacteria | 3 |
| *Rhodobacter sphaeroides 241* | Alphaproteobacteria | 3 |
| *Ralstonia eutropha H16* | Betaproteobacteria | 3 |
| *Mycoplasma penetrans HF-2* | Mollicutes | 3 |
| *Burkholderia xenovorans LB400* | Betaproteobacteria | 3 |
| *Novosphingobium aromaticivorans DSM 12444* | Alphaproteobacteria | 3 |
| *Methylobacillus flagellatus KT* | Betaproteobacteria | 3 |
| *Methanosarcina acetivorans C2A* | Methanomicrobia | 3 |
| *Salmonella enterica subsp enterica serovar Typhi str CT18* | Gammaproteobacteria | 3 |
| *Staphylococcus aureus subsp aureus NCTC 8325* | Bacillales | 2 |
| *Geobacter metallireducens GS-15* | Deltaproteobacteria | 2 |
| *Dehalococcoides ethenogenes 195* | Dehalococcoidetes | 2 |
| *Listeria monocytogenes EGD-e* | Bacillales | 2 |
| *Ehrlichia canis str Jake* | Alphaproteobacteria | 2 |
| *Candidatus Protochlamydia amoebophila UWE25* | Chlamydiales | 2 |
| *Nitrobacter hamburgensis X14* | Alphaproteobacteria | 2 |
| *Roseobacter denitrificans OCh 114* | Alphaproteobacteria | 2 |
| *Polaromonas sp JS666* | Betaproteobacteria | 2 |
| *Pseudomonas aeruginosa UCBPP-PA14* | Gammaproteobacteria | 2 |
| *Methanothermobacter thermautotrophicus str Delta H* | Methanobacteria | 2 |
| *Maricaulis maris MCS10* | Alphaproteobacteria | 2 |
| *Hahella chejuensis KCTC 2396* | Gammaproteobacteria | 2 |
| *Vibrio vulnificus YJ016* | Gammaproteobacteria | 2 |
| *Thiomicrospira crunogena XCL-2* | Gammaproteobacteria | 2 |
| *Sulfolobus tokodaii str 7* | Thermoprotei | 2 |
| *Pseudomonas entomophila L48* | Gammaproteobacteria | 2 |
| *Mycoplasma hyopneumoniae 232* | Mollicutes | 2 |
| *Syntrophus aciditrophicus SB* | Deltaproteobacteria | 2 |
| *Geobacillus kaustophilus HTA426* | Bacillales | 2 |
| *Nostoc sp PCC 7120* | Nostocales | 2 |
| *Burkholderia thailandensis E264* | Betaproteobacteria | 2 |
| *Mycoplasma genitalium G37* | Mollicutes | 2 |
| *Silicibacter pomeroyi DSS-3* | Alphaproteobacteria | 2 |
| *Mycoplasma mycoides subsp mycoides SC str PG1* | Mollicutes | 2 |
| *Lactobacillus plantarum WCFS1* | Lactobacillales | 2 |
| *Methanopyrus kandleri AV19* | Methanopyri | 2 |
| *Yersinia pseudotuberculosis IP 32953* | Gammaproteobacteria | 2 |
| *Neisseria meningitidis MC58* | Betaproteobacteria | 2 |
| *Dechloromonas aromatica RCB* | Betaproteobacteria | 2 |
| *Granulibacter bethesdensis CGDNIH1* | Alphaproteobacteria | 2 |
| *Lactobacillus sakei subsp sakei 23K* | Lactobacillales | 2 |
| *Treponema pallidum subsp pallidum str Nichols* | Spirochaetales | 2 |
| *Pseudomonas aeruginosa PAO1* | Gammaproteobacteria | 2 |
| *Pseudomonas syringae pv tomato str DC3000* | Gammaproteobacteria | 2 |
| *Listeria innocua Clip11262* | Bacillales | 2 |
| *Helicobacter hepaticus ATCC 51449* | Epsilonproteobacteria | 2 |
| *Pseudomonas fluorescens PfO-1* | Gammaproteobacteria | 2 |
| *Synechococcus sp WH 8102* | Chroococcales | 2 |
| *Leptospira borgpetersenii serovar Hardjo-bovis JB197* | Spirochaetales | 2 |
| *Bordetella parapertussis 12822* | Betaproteobacteria | 2 |
| *Corynebacterium glutamicum ATCC 13032* | Actinobacteridae | 2 |
| *Corynebacterium jeikeium K411* | Actinobacteridae | 2 |
| *Mesorhizobium sp BNC1* | Alphaproteobacteria | 2 |
| *Nitrosococcus oceani ATCC 19707* | Gammaproteobacteria | 2 |
| *Chlamydophila pneumoniae CWL029* | Chlamydiales | 2 |
| *Streptococcus pyogenes MGAS8232* | Lactobacillales | 2 |
| *Leptospira interrogans serovar Copenhageni str Fiocruz L1-130* | Spirochaetales | 2 |
| *Francisella tularensis subsp holarctica OSU18* | Gammaproteobacteria | 2 |
| *Burkholderia mallei ATCC 23344* | Betaproteobacteria | 2 |
| *Bacillus licheniformis ATCC 14580* | Bacillales | 2 |
| *Salinibacter ruber DSM 13855* | Sphingobacteria | 2 |
| *Shewanella sp MR-4* | Gammaproteobacteria | 2 |
| *Francisella tularensis subsp tularensis SCHU S4* | Gammaproteobacteria | 2 |
| *Helicobacter pylori J99* | Epsilonproteobacteria | 2 |
| *Desulfovibrio vulgaris subsp vulgaris str Hildenborough* | Deltaproteobacteria | 2 |
| *Mannheimia succiniciproducens MBEL55E* | Gammaproteobacteria | 2 |
| *Frankia alni ACN14a* | Actinobacteridae | 2 |
| *Methanosarcina barkeri str Fusaro* | Methanomicrobia | 2 |
| *Mesoplasma florum L1* | Mollicutes | 2 |
| *Methylococcus capsulatus str Bath* | Gammaproteobacteria | 2 |
| *Mycoplasma hyopneumoniae J* | Mollicutes | 2 |
| *Thermococcus kodakarensis KOD1* | Thermococci | 2 |
| *Propionibacterium acnes KPA171202* | Actinobacteridae | 2 |
| *Bacillus thuringiensis serovar konkukian str 97-27* | Bacillales | 2 |
| *Oceanobacillus iheyensis HTE831* | Bacillales | 2 |
| *Escherichia coli APEC O1* | Gammaproteobacteria | 2 |
| *Nanoarchaeum equitans Kin4-M* | Nanoarchaeum | 2 |
| *Bartonella quintana str Toulouse* | Alphaproteobacteria | 2 |
| *Pseudomonas fluorescens Pf-5* | Gammaproteobacteria | 2 |
| *Xanthomonas axonopodis pv citri str 306* | Gammaproteobacteria | 2 |
| *Natronomonas pharaonis DSM 2160* | Halobacteria | 2 |
| *Ehrlichia chaffeensis str Arkansas* | Alphaproteobacteria | 2 |
| *Anaplasma marginale str St Maries* | Alphaproteobacteria | 2 |
| *Streptococcus pyogenes SSI-1* | Lactobacillales | 2 |
| *Thermus thermophilus HB8* | Deinococci | 2 |
| *Desulfitobacterium hafniense Y51* | Clostridia | 2 |
| *Shigella flexneri 5 str 8401* | Gammaproteobacteria | 2 |
| *Staphylococcus aureus subsp aureus USA300* | Bacillales | 2 |
| *Frankia sp CcI3* | Actinobacteridae | 2 |
| *Vibrio fischeri ES114* | Gammaproteobacteria | 2 |
| *Shigella flexneri 2a str 301* | Gammaproteobacteria | 2 |
| *Borrelia garinii PBi* | Spirochaetales | 2 |
| *Yersinia pestis CO92* | Gammaproteobacteria | 2 |
| *Streptococcus pyogenes MGAS10750* | Lactobacillales | 2 |
| *Rhodoferax ferrireducens T118* | Betaproteobacteria | 2 |
| *Escherichia coli O157H7 str Sakai* | Gammaproteobacteria | 2 |
| *Rhizobium etli CFN 42* | Alphaproteobacteria | 2 |
| *Nitrosomonas europaea ATCC 19718* | Betaproteobacteria | 2 |
| *Colwellia psychrerythraea 34H* | Gammaproteobacteria | 2 |
| *Porphyromonas gingivalis W83* | Bacteroidetes | 2 |
| *Rickettsia conorii str Malish 7* | Alphaproteobacteria | 2 |
| *Lactococcus lactis subsp cremoris SK11* | Lactobacillales | 2 |
| *Burkholderia pseudomallei K96243* | Betaproteobacteria | 2 |
| *Shigella sonnei Ss046* | Gammaproteobacteria | 2 |
| *Brucella melitensis biovar Abortus 2308* | Alphaproteobacteria | 2 |
| *Brucella abortus biovar 1 str 9-941* | Alphaproteobacteria | 2 |
| *Xanthomonas oryzae pv oryzae MAFF 311018* | Gammaproteobacteria | 2 |
| *Silicibacter sp TM1040* | Alphaproteobacteria | 2 |
| *Shigella dysenteriae Sd197* | Gammaproteobacteria | 2 |
| *Dehalococcoides sp CBDB1* | Dehalococcoidetes | 2 |
| *Xylella fastidiosa Temecula1* | Gammaproteobacteria | 2 |
| *Neisseria meningitidis Z2491* | Betaproteobacteria | 2 |
| *Clostridium perfringens SM101* | Clostridia | 2 |
| *Shewanella sp MR-7* | Gammaproteobacteria | 2 |
| *Synechococcus sp JA-3-3Ab* | Chroococcales | 2 |
| *Psychrobacter arcticus 273-4* | Gammaproteobacteria | 1 |
| *Helicobacter pylori HPAG1* | Epsilonproteobacteria | 1 |
| *Yersinia pestis biovar Microtus str 91001* | Gammaproteobacteria | 1 |
| *Baumannia cicadellinicola str Hc Homalodisca coagulata* | Gammaproteobacteria | 1 |
| *Ehrlichia ruminantium str Gardel* | Alphaproteobacteria | 1 |
| *Helicobacter pylori 26695* | Epsilonproteobacteria | 1 |
| *Methanosphaera stadtmanae DSM 3091* | Methanobacteria | 1 |
| *Gluconobacter oxydans 621H* | Alphaproteobacteria | 1 |
| *Haloarcula marismortui ATCC 43049* | Halobacteria | 1 |
| *Salmonella typhimurium LT2* | Gammaproteobacteria | 1 |
| *Methanocaldococcus jannaschii DSM 2661* | Methanococci | 1 |
| *Yersinia pestis Nepal516* | Gammaproteobacteria | 1 |
| *Sulfolobus solfataricus P2* | Thermoprotei | 1 |
| *Mycobacterium leprae TN* | Actinobacteridae | 1 |
| *Escherichia coli 536* | Gammaproteobacteria | 1 |
| *Bacteroides fragilis YCH46* | Bacteroidetes | 1 |
| *Vibrio vulnificus CMCP6* | Gammaproteobacteria | 1 |
| *Prochlorococcus marinus str MIT 9312* | Prochlorales | 1 |
| *Xanthomonas oryzae pv oryzae KACC10331* | Gammaproteobacteria | 1 |
| *Picrophilus torridus DSM 9790* | Thermoplasmata | 1 |
| *Lactobacillus acidophilus NCFM* | Lactobacillales | 1 |
| *Bacillus cereus E33L* | Bacillales | 1 |
| *Burkholderia pseudomallei 1710b* | Betaproteobacteria | 1 |
| *Burkholderia cepacia AMMD* | Betaproteobacteria | 1 |
| *Pseudomonas syringae pv syringae B728a* | Gammaproteobacteria | 1 |
| *Streptococcus pyogenes MGAS5005* | Lactobacillales | 1 |
| *Thiobacillus denitrificans ATCC 25259* | Betaproteobacteria | 1 |
| *Mycoplasma pneumoniae M129* | Mollicutes | 1 |
| *Chlorobium tepidum TLS* | Chlorobia | 1 |
| *Buchnera aphidicola str Cc Cinara cedri* | Gammaproteobacteria | 1 |
| *Yersinia pestis KIM* | Gammaproteobacteria | 1 |
| *Shewanella frigidimarina NCIMB 400* | Gammaproteobacteria | 1 |
| *Rhizobium leguminosarum bv viciae 3841* | Alphaproteobacteria | 1 |
| *Staphylococcus epidermidis ATCC 12228* | Bacillales | 1 |
| *Wolbachia endosymbiont of Drosophila melanogaster* | Alphaproteobacteria | 1 |
| *Synechococcus elongatus PCC 7942* | Chroococcales | 1 |
| *Thermoplasma volcanium GSS1* | Thermoplasmata | 1 |
| *Sinorhizobium meliloti 1021* | Alphaproteobacteria | 1 |
| *Methanococcoides burtonii DSM 6242* | Methanomicrobia | 1 |
| *Clostridium perfringens str 13* | Clostridia | 1 |
| *Buchnera aphidicola str APS Acyrthosiphon pisum* | Gammaproteobacteria | 1 |
| *Rhodopseudomonas palustris HaA2* | Alphaproteobacteria | 1 |
| *Thermotoga maritima MSB8* | Thermotogales | 1 |
| *Bacillus halodurans C-125* | Bacillales | 1 |
| *Mycobacterium sp MCS* | Actinobacteridae | 1 |
| *Deinococcus radiodurans R1* | Deinococci | 1 |
| *Bacteroides thetaiotaomicron VPI-5482* | Bacteroidetes | 1 |
| *Shigella flexneri 2a str 2457T* | Gammaproteobacteria | 1 |
| *Borrelia burgdorferi B31* | Spirochaetales | 1 |
| *Staphylococcus epidermidis RP62A* | Bacillales | 1 |
| *Rhodococcus sp RHA1* | Actinobacteridae | 1 |
| *Streptococcus pyogenes MGAS2096* | Lactobacillales | 1 |
| *Bacteroides fragilis NCTC 9343* | Bacteroidetes | 1 |
| *Oenococcus oeni PSU-1* | Lactobacillales | 1 |
| *Synechococcus sp CC9605* | Chroococcales | 1 |
| *Burkholderia sp 383* | Betaproteobacteria | 1 |
| *Chlamydophila caviae GPIC* | Chlamydiales | 1 |
| *Bordetella pertussis Tohama I* | Betaproteobacteria | 1 |
| *Staphylococcus aureus subsp aureus MSSA476* | Bacillales | 1 |
| *Streptococcus agalactiae A909* | Lactobacillales | 1 |
| *Streptomyces avermitilis MA-4680* | Actinobacteridae | 1 |
| *Pelobacter carbinolicus DSM 2380* | Deltaproteobacteria | 1 |
| *Thermoplasma acidophilum DSM 1728* | Thermoplasmata | 1 |
| *Mycoplasma hyopneumoniae 7448* | Mollicutes | 1 |
| *Anaeromyxobacter dehalogenans 2CP-C* | Deltaproteobacteria | 1 |
| *Gloeobacter violaceus PCC 7421* | Gloeobacteria | 1 |
| *Pyrococcus horikoshii OT3* | Thermococci | 1 |
| *Sphingopyxis alaskensis RB2256* | Alphaproteobacteria | 1 |
| *Ehrlichia ruminantium str Welgevonden* | Alphaproteobacteria | 1 |
| *Lactobacillus brevis ATCC 367* | Lactobacillales | 1 |
| *Xanthomonas campestris pv campestris str ATCC 33913* | Gammaproteobacteria | 1 |
| *Pyrococcus furiosus DSM 3638* | Thermococci | 1 |
| *Moorella thermoacetica ATCC 39073* | Clostridia | 1 |
| *Bacillus anthracis str Sterne* | Bacillales | 1 |
| *Streptomyces coelicolor A32* | Actinobacteridae | 1 |
| *Pseudoalteromonas atlantica T6c* | Gammaproteobacteria | 1 |
| *Thermosynechococcus elongatus BP-1* | Chroococcales | 1 |
| *Xylella fastidiosa 9a5c* | Gammaproteobacteria | 1 |
| *Staphylococcus aureus subsp aureus N315* | Bacillales | 1 |
| *Chromobacterium violaceum ATCC 12472* | Betaproteobacteria | 1 |
| *Haemophilus somnus 129PT* | Gammaproteobacteria | 1 |

Species and taxonomic classes for the contigs in the random dataset. The number of contigs for each species in the dataset is also shown.

**Supplementary table 2**

|  | ORFs | Assigned | Same | Different | Contigs | Assigned | Same | Different |
| --- | --- | --- | --- | --- | --- | --- | --- | --- |
| Planctomycetacia | 449 | 47 | 47 | 0 | 100 | 10 | 10 | 0 |
| Dehalococcoidetes | 486 | 307 | 305 | 2 | 100 | 85 | 85 | 0 |
| Methanopyri | 424 | 5 | 0 | 5 | 100 | 2 | 0 | 2 |

Results of the phylogenetic assignment of 100 sequences from rare taxa.

**Supplementary table 3**

| Other | 165 |
| --- | --- |
| Chlorobia | 73 |
| Clostridia | 88 |
| Beta-proteobacteria | 120 |
| Sphingobacteria | 155 |
| Bacteroidales | 159 |
| Delta-proteobacteria | 599 |
| Flavobacteria | 671 |
| Epsilon-proteobacteria | 1657 |
| Alpha-proteobacteria | 2354 |
| Gamma-proteobacteria | 3791 |

| Other | 746 |
| --- | --- |
| Nostocales | 130 |
| Planctomycetacia | 344 |
| Flavobacteria | 526 |
| Chloroflexales | 542 |
| Actinobacteridae | 545 |
| Sphingobacteria | 571 |
| Gamma-proteobacteria | 770 |
| Delta-proteobacteria | 1052 |
| Beta-proteobacteria | 1304 |
| Alpha-proteobacteria | 2835 |

Whale fall 1 Farm Soil

| Other | 55 |
| --- | --- |
| Spirochaetales | 16 |
| Alpha-proteobacteria | 16 |
| Epsilon-proteobacteria | 18 |
| Bacteroidales | 20 |
| Gamma-proteobacteria | 41 |
| Bacillales | 97 |
| Lactobacillales | 188 |
| Methanobacteria | 248 |
| Actinobacteridae | 1212 |
| Clostridia | 1233 |

| Other | 490 |
| --- | --- |
| Chlorobia | 90 |
| Delta-proteobacteria | 125 |
| Thermoprotei | 126 |
| Sphingobacteria | 151 |
| Actinobacteridae | 154 |
| Chroococcales | 492 |
| Beta-proteobacteria | 802 |
| Prochlorales | 934 |
| Flavobacteria | 1155 |
| Gamma-proteobacteria | 3932 |
| Alpha-proteobacteria | 11925 |

Human gut Sargasso Sea

Number of assignments (class taxonomic rank) for the contigs of different metagenomes.

**Supplementary table 4**

| Whale fall 1 | Farm soil | Gut | Sargasso |
| --- | --- | --- | --- |
| 29.4/17.6 **(39.9%)** | 56.9/29.1 **(48.8%)** | 15.4/10.8 **(29.8%)** | 52.3 **(22.6%)** |

Length of sequence analyzed (Mbp), length of sequence with homologues (Mbp), and, in parenthesis, percentage of sequence for which no homologues could be found. Farm soil metagenome is the one with the highest percentage of DNA for which no homologs could be found. This metagenome is also the most diverse in terms of taxonomic composition (see text).

**Supplementary table 5**

| Whale Fall1 | Human gut | Farm soil | Sargasso Sea |
| --- | --- | --- | --- |
| 14:Alphaproteobacteria  Gammaproteobacteria | 10: Clostridia  Lactobacillales | 13: Flavobacteria  Sphingobacteria | 20:Gammaproteobacteria  Alphaproteobacteria |
| 9: Flavobacteria  Sphingobacteria | 5: Bacillales  Clostridia | 10:Betaproteobacteria  Alphaproteobacteria | 15:Gammaproteobacteria  Betaproteobacteria |
| 9:Epsilonproteobacteria  Gammaproteobacteria | 4:Actinobacteridae  Lactobacillales | 8:Gammaproteobacteria  Alphaproteobacteria | 15: Flavobacteria  Alphaproteobacteria |
| 5: Betaproteobacteria  Gammaproteobacteria | 3:Methanobacteria  Methanococci | 8: Flavobacteria  Bacteroidales | 10: Flavobacteria  Alphaproteobacteria |
| 4: Bacteroidales  Flavobacteria | 2:Actinobacteridae  Clostridia | 6: Betaproteobacteria  Gammaproteobacteria | 7: Flavobacteria  Sphingobacteria |

Pairs of taxa involved in possible HGT events (the direction of the event cannot be determined). The number of events is also indicated. Only the five most abundant instances are shown.

**Supplementary table 6**

| Difference  in methods | Number of contigs | clust 0.3 | clust 0.4 | clust 0.5 | clust 0.55 | clust 0.6 | clust 0.65 | clust 0.7 |
| --- | --- | --- | --- | --- | --- | --- | --- | --- |
| 0 | 1327 | 5 | 12 | 48 | 84 | 126 | 195 | 300 |
| 1 | 1223 | 23 | 43 | 75 | 120 | 169 | 228 | 351 |
| 2 | 654 | 38 | 61 | 112 | 141 | 195 | 239 | 290 |
| 3 | 384 | 63 | 96 | 128 | 148 | 181 | 214 | 251 |
| 4 | 241 | 67 | 92 | 127 | 148 | 171 | 190 | 207 |
| 5 | 125 | 49 | 64 | 76 | 85 | 94 | 102 | 113 |
| 6 | 44 | 12 | 21 | 29 | 33 | 35 | 37 | 41 |

Number of contigs proposed to contain a compositional transition, according to different values the clustering correlation parameter. The first column indicates the difference in the number of methods that propose consecutive ORFs as being horizontally transferred. For instance, if just one method predicts an ORF as transferred, but the next is predicted by four, the difference in methods is equal to three. The higher the value of this difference, the more likely it is that the contig shows a compositional transition. The second column indicates how many contigs have a given difference in method. Remaining columns indicate how many of these contigs are predicted as containing a compositional transition, for different values of the clustering correlation parameter.
