# Supplementary material for: Estimating the extent of horizontal gene transfer in metagenomic sequences
Source: BMC Genomics. 2008 Mar 24;9:136. doi: 10.1186/1471-2164-9-136 (PMC2324111; doi:10.1186/1471-2164-9-136)
Supplement: Additional file 4 — Example of the phylogenetic method. Three examples of the procedure for the taxonomic assignment by the phylogenetic method. Phylogenetic trees have been created with the homologues found for three different metagenomic ORFs. Homologues are coloured according to their taxonomic affiliation. The position of the query metagenomic ORF is signalled by the black arrows in the trees. The tables at the bottom of the trees show the sorted list of the homologues and their distances to the query ORF. A) The query ORF is monophyletic with the Epsilon-proteobacteria taxon. Therefore, the lowest distance scores are those for the homologues belonging to that taxon, and the query ORF is automatically assigned to it. B) The ORF is closely related to alpha-proteobacteria, but there are some homologues belonging to that taxa that are distantly related. Nevertheless, the average distance score for alpha-proteobacteria is more than 15% lower than the average distance for any other taxon, thus allowing its assignment to it. C) In this case, neither the ORF is monophyletic with any taxon, nor the average distance scores allow the assignment, and the query ORF remains unassigned. [file 1471-2164-9-136-S4.ppt]

## Slide 1
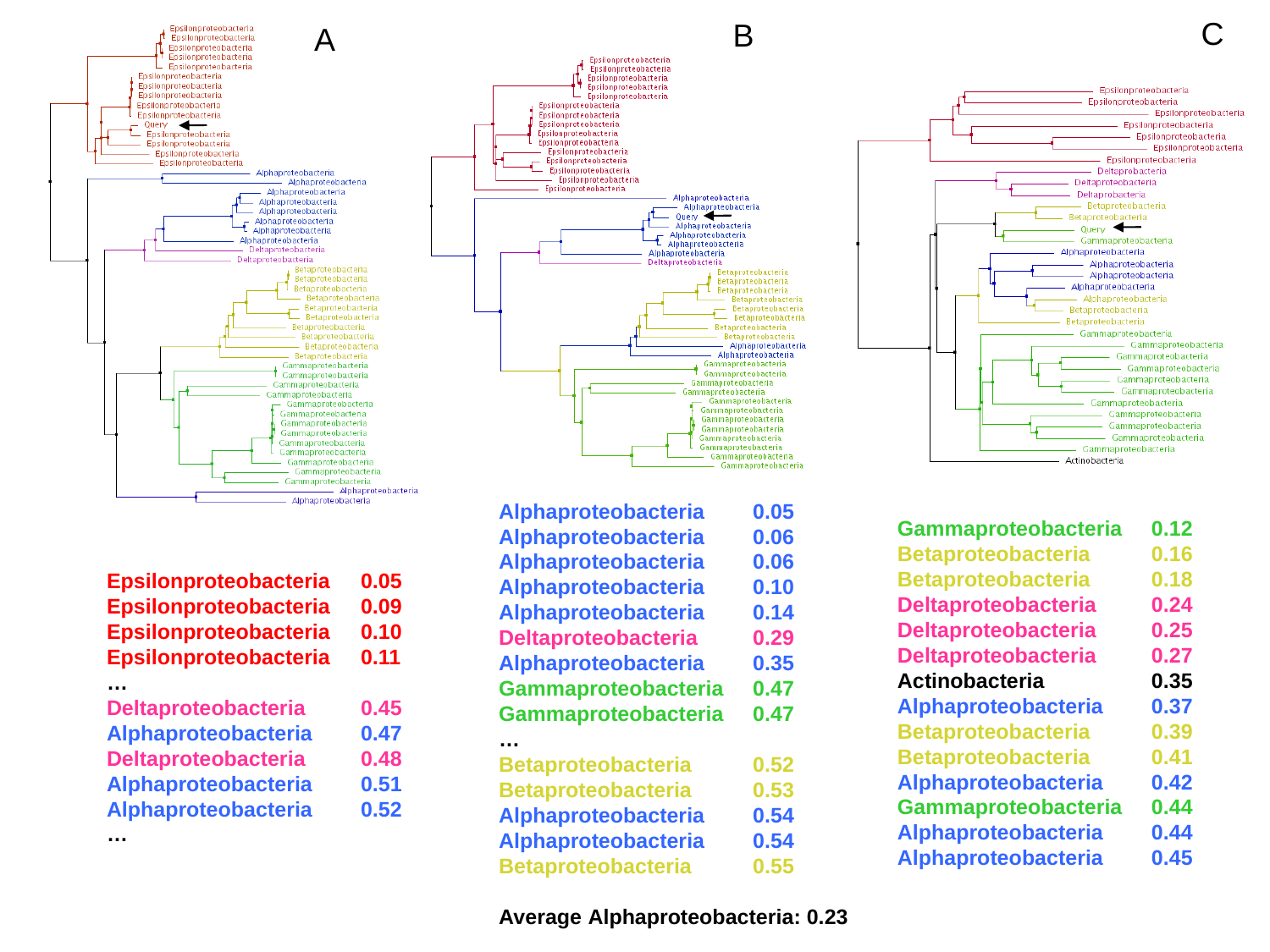

C
B
A
Alphaproteobacteria	0.05
Alphaproteobacteria	0.06
Alphaproteobacteria	0.06
Alphaproteobacteria	0.10
Alphaproteobacteria	0.14
Deltaproteobacteria	0.29
Alphaproteobacteria	0.35
Gammaproteobacteria	0.47
Gammaproteobacteria	0.47
…
Betaproteobacteria	0.52
Betaproteobacteria	0.53
Alphaproteobacteria	0.54
Alphaproteobacteria	0.54
Betaproteobacteria	0.55
Average Alphaproteobacteria: 0.23
Gammaproteobacteria	0.12
Betaproteobacteria	0.16
Betaproteobacteria	0.18
Deltaproteobacteria	0.24
Deltaproteobacteria	0.25
Deltaproteobacteria	0.27
Actinobacteria	0.35
Alphaproteobacteria	0.37
Betaproteobacteria	0.39
Betaproteobacteria	0.41
Alphaproteobacteria	0.42
Gammaproteobacteria	0.44
Alphaproteobacteria	0.44
Alphaproteobacteria	0.45
Epsilonproteobacteria	0.05
Epsilonproteobacteria	0.09
Epsilonproteobacteria	0.10
Epsilonproteobacteria	0.11
…
Deltaproteobacteria	0.45
Alphaproteobacteria	0.47
Deltaproteobacteria	0.48
Alphaproteobacteria	0.51
Alphaproteobacteria	0.52
…
