# Supplementary material for: Estimating the extent of horizontal gene transfer in metagenomic sequences
Source: BMC Genomics. 2008 Mar 24;9:136. doi: 10.1186/1471-2164-9-136 (PMC2324111; doi:10.1186/1471-2164-9-136)
Supplement: Additional file 7 — Full analysis of a single contig. Analysis of contig AAFY01000115 from the whale fall metagenome. Homology searches indicate that the contig contains three ORFs. A: The compositional method identifies a transition in the contig. The first two ORFs show a similar composition, but the third differs. In addition, the second ORF is a transposase, which supports the idea of a probable HGT. B: Taxonomic assignment provides a result for the two first ORFs (alpha-proteobacteria), but not for the third. The third ORF finds only one distant homologue (27% identity) with a gamma-proteobacteria (Acinetobacter sp.), and therefore an assignment cannot be made. As a result, this contig is recognised as a probable HGT only by the compositional method. [file 1471-2164-9-136-S7.ppt]

## Slide 1
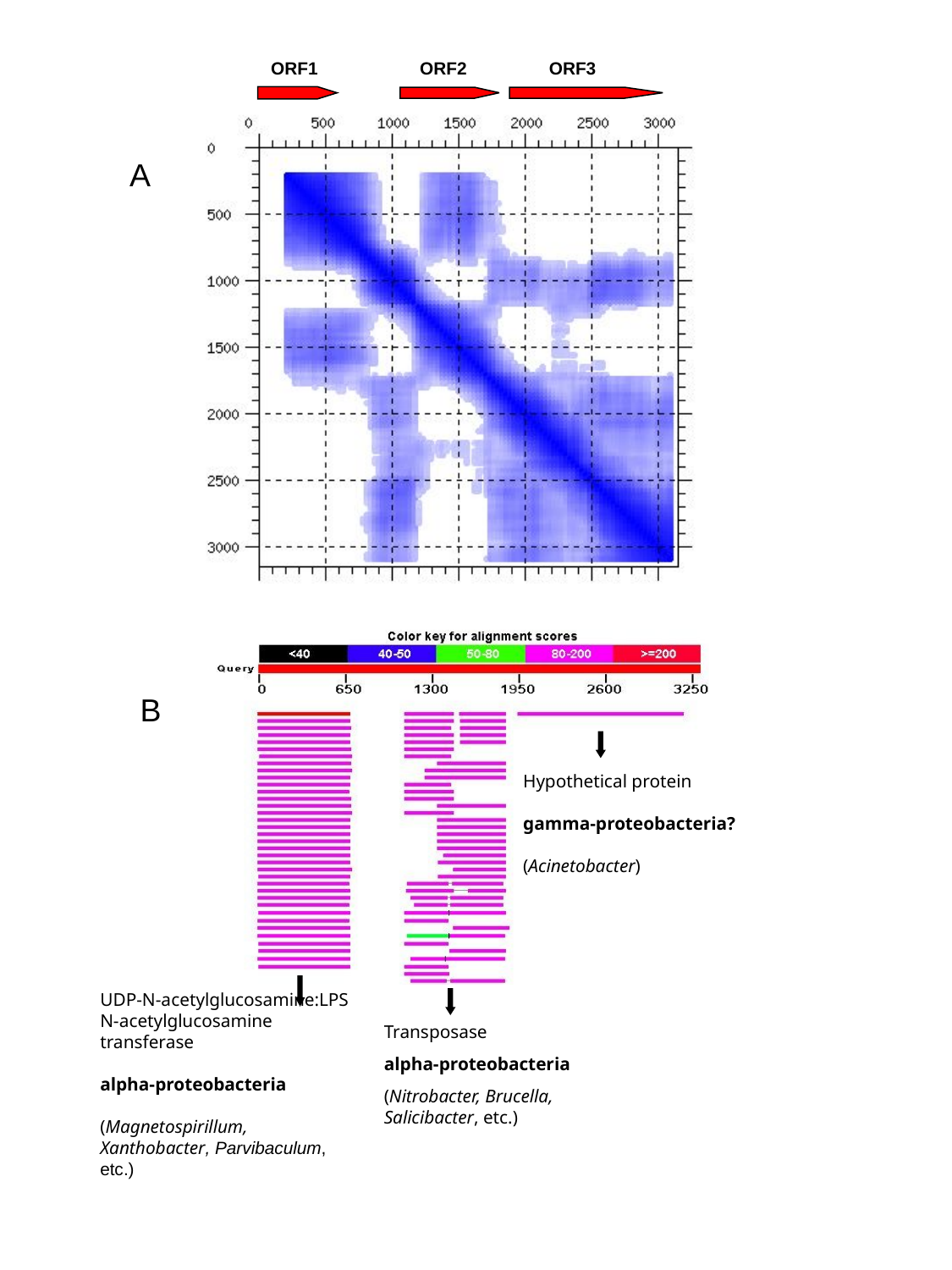

ORF1
ORF2
ORF3
A
Hypothetical protein
gamma-proteobacteria?
(Acinetobacter)
UDP-N-acetylglucosamine:LPS N-acetylglucosamine transferase
alpha-proteobacteria
(Magnetospirillum, Xanthobacter, Parvibaculum, etc.)
Transposase
alpha-proteobacteria
(Nitrobacter, Brucella, Salicibacter, etc.)
B
